# A virulence factor as a therapeutic: the probiotic *Enterococcus faecium* SF68 arginine deiminase inhibits innate immune signaling pathways

**DOI:** 10.1080/19490976.2022.2106105

**Published:** 2022-08-03

**Authors:** Fereshteh Ghazisaeedi, Jochen Meens, Bianca Hansche, Sven Maurischat, Peter Schwerk, Ralph Goethe, Lothar H. Wieler, Marcus Fulde, Karsten Tedin

**Affiliations:** a Department of Veterinary Medicine, Institute of Microbiology and Epizootics, Centre for Infection Medicine, Free University of Berlin, Berlin, Germany; b Institute for Microbiology, University of Veterinary Medicine, Hannover, Germany; c Sanofi-AventisGmbH, Berlin, Germany; d German Federal Institute for Risk Assessment (BfR), Berlin, Germany; e Robert Koch Institute, Berlin, Germany

**Keywords:** *Enterococcus faecium* SF68, probiotics, NF-κB, intestinal epithelial cells, arginine deiminase, innate immune response

## Abstract

The probiotic bacterial strain *Enterococcus faecium* SF68 has been shown to alleviate symptoms of intestinal inflammation in human clinical trials and animal feed supplementation studies. To identify factors involved in immunomodulatory effects on host cells, *E. faecium* SF68 and other commensal and clinical *Enterococcus* isolates were screened using intestinal epithelial cell lines harboring reporter fusions for NF-κB and JNK(AP-1) activation to determine the responses of host cell innate immune signaling pathways when challenged with bacterial protein and cell components. Cell-free, whole-cell lysates of *E. faecium* SF68 showed a reversible, inhibitory effect on both NF-κB and JNK(AP-1) signaling pathway activation in intestinal epithelial cells and abrogated the response to bacterial and other Toll-like receptor (TLR) ligands. The inhibitory effect was species-specific, and was not observed for *E. avium, E. gallinarum*, or *E. casseliflavus*. Screening of protein fractions of *E. faecium* SF68 lysates yielded an active fraction containing a prominent protein identified as arginine deiminase (ADI). The *E. faecium* SF68 *arcA* gene encoding arginine deiminase was cloned and introduced into *E. avium* where it conferred the same NF-_κ_B inhibitory effects on intestinal epithelial cells as seen for *E. faecium* SF68. Our results indicate that the arginine deiminase of *E. faecium* SF68 is responsible for inhibition of host cell NF-κB and JNK(AP-1) pathway activation, and is likely to be responsible for the anti-inflammatory and immunomodulatory effects observed in prior clinical human and animal trials. The implications for the use of this probiotic strain for preventive and therapeutic purposes are discussed.

## Introduction

The alarming increase in bacterial antibiotic resistance worldwide has lead to a fundamental reassessment of the use of antibiotics in both therapeutic and prophylactic applications.^1^ Increased restrictions in the therapeutic application of antibiotics in human and veterinary medicine, and the ban of antibiotics as growth promoters in livestock, have heightened the need for alternatives to antibiotics in both human and animal health and the food industry.^2,3^ As a result of these developments, increased interest and research has focused on the use of beneficial microbes as both prophylactics and therapeutics in human and animal health.^4,5^ Probiotic microorganisms have been found to confer health benefits to the host through modification and modulation of microbiota, alleviation of dysbiosis, niche shifts in favor of colonization by beneficial microorganisms, exclusion of potential pathogens, stimulation or inhibition of the immune system, and dampening of pro-inflammatory properties of altered, damaged, or infected cells.^6,7^ Likewise, both natural and engineered probiotic microbes have been reported to enhance absorption and digestion in the gut, increase antibacterial activities and elimination of pathogens, and have been used for the prevention and treatment of diarrhea and reduction of inflammatory responses in chronic inflammatory diseases, *e.g*. inflammatory bowel disease, Crohn’s disease, cancers, and enteric infections.^8,9^

*Enterococcus faecium* SF68 (NCIMB 10415), is an endogenous, intestinal commensal isolate, and a well-characterized member of the lactic acid bacteria (LAB), which has been authorized for use as a probiotic in pharmaceutical preparations and food supplements in humans and animals.^10–13^ As with many other LAB, the strain metabolizes carbon sources through fermentation and substrate level phosphorylation reactions rather than oxidative phosphorylation for generation of ATP.^8,9,14^ Glycolysis and fermentation of sugars generates acetate and lactic acid, providing a growth advantage for LAB which are generally resistant to low pH.^8,15^ Furthermore, production of ATP through catabolism of arginine by the arginine deiminase pathway (ADI) generates ammonia as an end-product contributing to survival in acidified environments, although *E. faecium* is generally not thought to use arginine as a source of ATP.^8^

*E. faecium* strain SF68 has been shown to mitigate symptoms of human and animal intestinal inflammation. In humans, it is used as a treatment for diarrhea, particularly in cases of antibiotic-associated diarrhea. Positive effects of *E. faecium* SF68 on the reduction and duration of diarrhea have also been demonstrated in animals.^12,13^ Despite the generally positive effects reported for *E. faecium* SF68, there are conflicting reports concerning the beneficial effects of this probiotic strain. Results of some *in vivo* trials in different host species supplemented with *E. faecium* SF68 found no significant beneficial effects or even adverse effects, such as a higher bacterial loads and shedding of enteric pathogens including *Salmonella* serovar Typhimurium in animal infection studies.^16–20^

In a number of *in vivo* studies in post-weaning piglets treated with *E. faecium* SF68, we and others observed an apparent immune dysregulation in intestinal tissues, including lower serum total IgG and fecal IgA, and an attenuation of CD8^+^ intraepithelial lymphocyte populations.^16,17^ Post-weaning piglets supplemented with *E. faecium* SF68 followed by a challenge with *Salmonella* Typhimurium aggravated the course of infection, characterized by higher rates of dissemination and colonization of the pathogen in host organs, elevated fecal shedding of *Salmonella*, and reduced or delayed proliferative responses to *Salmonella* antigens by peripheral blood mononuclear cells.^19–21^ Furthermore, in an animal feeding trial, we observed significantly reduced expression of immune-associated genes of intestinal and associated lymphoid tissues in post-weaning piglets supplemented with this strain.^21^

As the factors and mechanism(s) involved in the probiotic effects of *E. faecium* SF68 are not very well known, the aim of this study was the identification and characterization of possible immunomodulatory factors involved in the observed effects on immune-associated gene expression in previous animal trials in post-weaning piglets, with the larger goal of explaining prior clinical studies reporting beneficial, probiotic effects on recovery from intestinal inflammation and diarrhea in humans and animals.

## Results

### E. faecium SF68 inhibits NF-κB activity of intestinal epithelial cells

As our previous *in vivo* studies indicated an inhibitory effect on both pro- and anti-inflammatory immune-associated gene expression by *E. faecium* SF68,^21^ we focused on the activation status of nuclear factor-κB (NF-κB), a central host cell transcription factor involved in regulation of genes involved in growth and metabolism, as well as innate immune responses of many cell types, including intestinal epithelial cells.^22,23^ The NF-κB signaling pathway consists of the NF-κB proteins NF-κB1(p50), RelA(p65), and c-Rel, among others. In resting cells, NF-κB is retained in an inactive form in the cytosol bound by the inhibitor of κB (I_k_B), which must first be phosphorylated followed by ubiquitylation and degradation by the proteosome, resulting in release (activation) of NF-κB which then translocates to the nucleus to activate NF-κB-dependent gene expression.^23,24^

**Figure 1.**
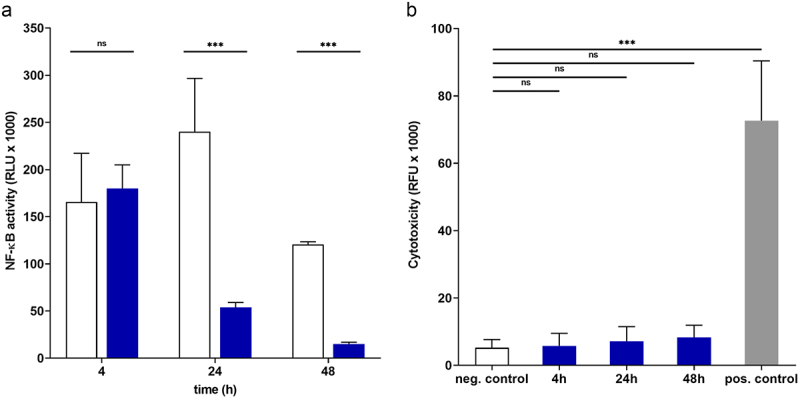
The probiotic *E. faecium* SF68 inhibits NF-κB activity in intestinal epithelial cells. (a) The porcine, intestinal epithelial cell line IPEC-J2 harboring an NF-κB responsive luciferase reporter fusion (IPEC-J2/K6) was incubated for the times indicated in the presence of gentamicin-killed, intact *E. faecium* SF68 at a multiplicity of infection (MOI) of 100:1 bacteria: host cells. The basal levels of NF-κB activity are indicated by open bars, and the treated cells with filled bars. (b) Cytotoxicity assays performed in parallel to the NF-kκB assays shown in panel A. The results shown are representative of at least three, independent assays.

To determine the possible effects of *E. faecium* SF68 on NF-κB activation, we initially performed co-incubation experiments with various concentrations of *E. faecium* SF68 and a porcine, intestinal epithelial cell line (IPEC-J2/K6) harboring an NF-κB transcriptional reporter responsive to NF-κB activation. In preliminary studies, it was not possible to treat cells with viable *E. faecium* SF68 for times longer than 4 h, due to the rapid acidification of the cell culture media as a result of bacterial metabolism. To avoid this complication, we first treated *E. faecium* SF68 with gentamicin at concentrations resulting in bacterial killing, but which left the bacteria intact. As shown in Figure 1(a), treatment of host cells with killed but intact bacteria resulted in an initial activation of NF-κB, as expected, but longer incubations showed an inhibition of NF-κB activity to levels below the basal levels of control, untreated cells. Notably, the multiplicity of infection (MOI) of bacterial cells:host cells in these assays (100:1) are at the lower range observed for *E. faecalis* microcolonies adherent to the intestinal wall *in vivo* studies in mice.^25^ Furthermore, we observed no acidification of the cell culture medium using killed *E. faecium* SF68, indicating that the NF-κB inhibition was not the result of accumulation of acidic metabolites derived from glycolysis such as acetate or lactate. Likewise, incubation of host cells with the killed but intact bacteria did not show indications of host cell cytoxicity (Figure 1(b)).

The results shown in Figure 1 indicated that some factor(s) present in killed but otherwise intact *E. faecium* SF68 was capable of inhibition of NF-κB activity. Due to the long incubation times in the presence of gentamicin required in order to achieve sufficient bacterial killing (≥4 h), it was possible that protein turnover/degradation during the treatment resulted in low levels of the putative inhibitory factor of NF-κB activity. In order to avoid the complication of bacterial metabolism of the cell culture medium and possible loss of putative protein factors due to degradation, we therefore chose to treat the host cells with cell-free, bacterial lysates of *E. faecium* SF68. In order to standardize the assays, the total protein concentrations of the bacterial lysates were determined, and corrected for the efficiency of lysis to yield CFU equivalents/µg lysate (see Materials and Methods). In all assays, 5 µg of these cell-free, bacterial lysates were used in the cell culture co-incubation assays, corresponding to an approximate MOI of 300:1 bacterial CFU equivalents:host cells, a ratio within the same range of *E. faecalis* microcolonies to enterocytes observed *in vivo*.^25^

**Figure 2.**
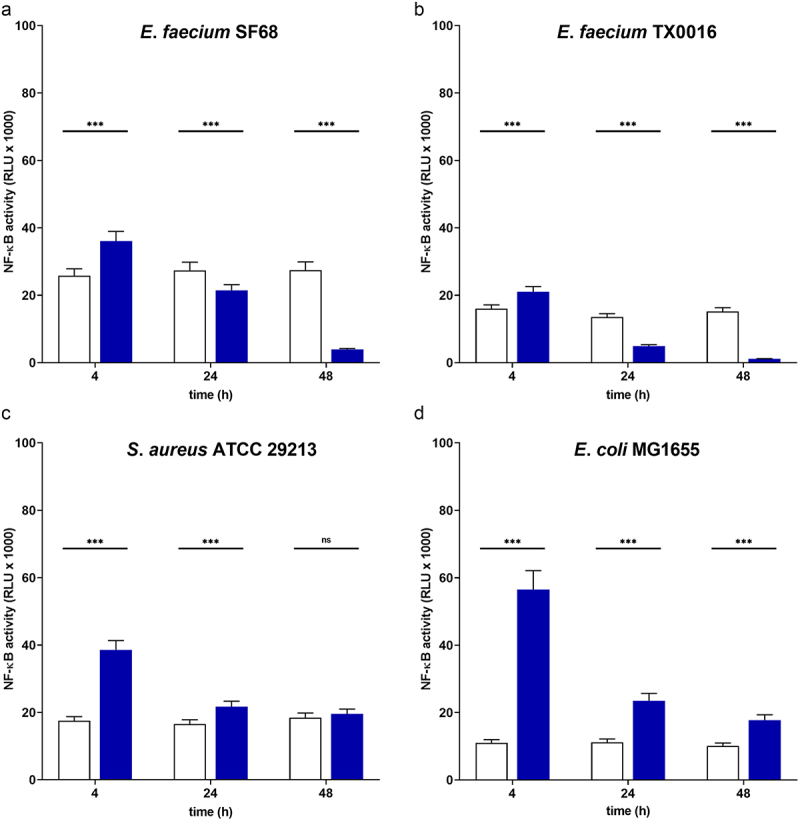
The probiotic *E. faecium* SF68 and pathogenic *E. faecium* TX0016 strains show severe inhibition of NF-κB activity in intestinal epithelial cells. The IPEC-J2/K6 cell line was incubated for the times indicated in the presence of cell-free, whole cell bacterial lysates of (a) *E. faecium* SF68, (b) *E. faecium* TX0016, (c) *S. aureus* ATCC 29213, or (d) *E. coli* K-12 strain MG1655. In all panels, the basal levels of NF-κB activity are indicated by open bars, and the treated cells with filled bars. The results shown are representative of at least three, independent assays.

As shown in Figure 2(a), treatment of host cells with bacterial lysates of *E. faecium* SF68 showed the same time-dependent reduction in NF-κB activation, to levels below that of untreated, cells within 24 h of incubation. To determine whether this effect was specific for *E. faecium* SF68 or a general feature of enterococcal strains, we also performed the same treatments using cell-free, bacterial lysates of a well-characterized pathogenic strain of *E. faecium*, TX0016. As seen in Figure 2(b), we observed the same time-dependent inhibition of NF-κB activity. In contrast, treatment of host cells with bacterial lysates of other genera including *Staphylococcus aureus* ATCC 29213 or *Escherichia coli* K-12 MG1655 showed an initial increase in NF-κB activation 4 h post-treatment, but which declined to the basal levels of NF-κB activity at later time points (Figure 2 (c) and (d), resp.).

### E. faecium SF68 treatment inhibits host cell proliferation but is not cytotoxic

Despite no obvious cytotoxic effects on the host cells such as rounding-up or detachment of monolayers during treatment with the bacterial lysates, a possible explanation for the loss of NF-κB activity could have been cytotoxic effects not immediately visible during the course of the treatments. We therefore examined the treated cell cultures by microscopy and after staining cells with antibodies targeting the cell proliferation marker Ki67.^26,27^ In addition to its role in regulation of immune responses, NF-κB also regulates genes involved in epithelial cell homeostasis and proliferation, examples of which can be found at http://www.bu.edu/nf-kb/gene-resources/target-genes.^28–30^ Cultures of untreated cells showed intact monolayers with isolated cells in various stages of proliferation, as judged by the degree of staining with Ki67. In contrast, cells challenged with *E. faecium* SF68 cell lysates clearly showed intact monolayers, but only rare cells showed staining with anti-Ki67 antibodies, indicating the majority of cells had ceased to proliferate (see supplementary Figure S1).

While visual and microscopic inspection of the cell monolayers showed intact monolayers with no obvious morphological changes suggestive of cell death, it remained possible the *E. faecium* SF68 cell lysates induced cytotoxic effects not immediately obvious by visual inspection. We therefore performed a number of cell cytotoxicity and viability assays with cell cultures treated with the cell-free bacterial lysates. Cell viability assays comparing cells treated with the *E. faecium* SF68 lysates and untreated cells, indicated no major effects on cell viability during the first 24 h of incubation (supplementary Figure S2A). An approximately 30% reduction in viability of host cells was observed after 48 h of co-incubation with the *E. faecium* SF68 lysates, but which was attributable to the severely reduced NF-κB basal activity to only around 10% that of untreated, resting cells as seen in Figures 1 and 2. In addition, cells treated with *E. faecium* SF68 lysates showed no increase in lactate dehydrogenase (LDH) release into the cell culture media, indicating no loss of cell membrane integrity, an indicator of cell cytotoxicity (supplementary Figure S2B). Furthermore, co-treatment of cells with the caspase inhibitor Z-VAD-FMK did not improve the NF-κB activation levels (supplementary Figure S2C), indicating that the *E. faecium* SF68 lysates were not involved in activation of apoptosis, as previously reported for other *E. faecium* and *E. faecalis* isolates.^31–34^ Finally, it has previously been reported that *E. faecium* and *Streptococcus* spp. isolates produce cytotoxic levels of hydrogen peroxide leading to an oxidative stress-associated death of host cells.^35–37^ We therefore also determined the NF-κB activation levels of cells treated with *E. faecium* SF68 lysates in the presence of catalase, which would detoxify any hydrogen peroxide present in the medium. The presence of catalase did not affect the NF-κB inhibition by the *E. faecium* lysates (supplementary Figure S2D), indicating that the inhibitory effects on NF-κB activation was not due to production of hydrogen peroxide by the bacterial lysates.

### Enterococcal inhibition of NF-κB activity is species-specific

**Figure 3.**
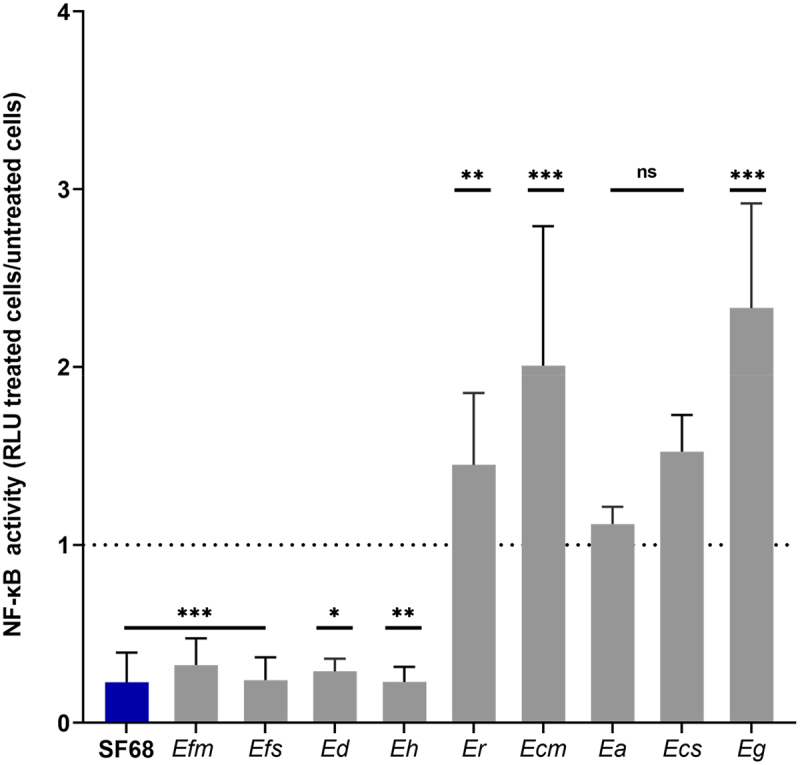
Inhibition of NF-κB activity is *Enterococcus* species-specific. Confluent monolayers of the IPEC-J2/K6 NF-κB reporter cell line was incubated in the presence of 5 µg of cell-free, bacterial lysate of either *E. faecium* SF68 (blue bar) or other representative *Enterococcus* species (gray bars) as indicated below the graph for 24 h followed by determination of NF-κB (luciferase) activity. The dotted line indicates the basal NF-κB activity of untreated cells determined in parallel. SF68, *E. faecium* SF68; Efm, *E. faecium* TX1310; Efs, *E. faecalis* ATCC 29212; Ed, *E. durans* IMT38978; Eh, *E. hirae* ATCC 9790; Er, *E. raffinosus* IMT23827; Ecm, *E. cecorum* IMT19051; Ea, *E. avium* IMT39925; Ecs, *E. casseliflavus* IMT39928; Eg, *E. gallinarum* IMT12257. The results shown are the averages of at least three, independent assays for each *Enterococcus* strain, and are reported as the relative NF-κB activity of treated cells compared to untreated, control cells from the same experiment. See supplementary Figure. S3 for additional isolates of all species and Table S1 for additional strain information.

The inhibitory effects of bacterial lysates on NF-κB activation shown in Figure 2 appeared to be bacterial genera-specific, characteristic only for the *Enterococcus faecium* isolates tested, and not a general response of the cell line to treatments with bacterial cell lysates. To explore this observation further, we screened additional clinical and commensal isolates of *E. faecium* as well as isolates of other *Enterococcus* species to determine whether the inhibitory effects were a general characteristic of the genus *Enterococcus*. We tested a total of 48 isolates of *E. faecium, E. avium, E. casseliflavus, E. cecorum, E. durans, E. faecalis, E. gallinarum, E. hirae*, and *E. raffinosus*. As shown in Figure 3, cell-free lysates of representative isolates of *E. faecium, E. faecalis, E. durans* and *E. hirae* isolates showed the same inhibitory effects on NF-κB activation of intestinal epithelial cells, whereas isolates of *E. avium, E. casseliflavus, E. cecorum, E. gallinarum*, and *E. raffinosus* showed either unchanged or elevated NF-κB activity. Similar results were found for additional isolates of these *Enterococcus* species (supplementary Figure S3 and Table S2). These results indicated that the inhibition of NF-κB activation was a characteristic which the probiotic *E. faecium* SF68 strain shared with other *Enterococcus* species, including both commensal and pathogenic strains, but which was not a general feature of all *Enterococcus* species.

### The inhibitory effect of E. faecium SF68 on NF-κB activation levels is independent of the host cell background

**Figure 4.**
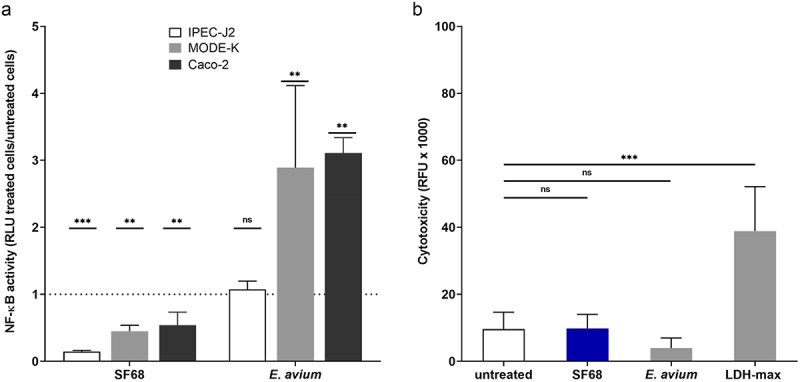
*E. faecium* SF68 inhibition of NF-κB activity is independent of the host species origin of intestinal epithelial cells. (a) Confluent monolayers of NF-κB-luciferase reporter cell lines of porcine IPEC-J2 (open bars), human Caco-2 (gray bars) or murine MODE-K (black bars) intestinal epithelial cells were treated with 5 µg of total protein of cell-free, bacterial lysates of either *E. faecium* SF68 or *E. avium* IMT39925, for 24 h prior to determination of NF-κB activity. The results shown are reported as the averages of the ratios of NF-κB activity of treated/untreated cells from at least three, independent assays. The dotted line indicates the normalized, average basal NF-κB activity determined for untreated cells. (b) Cytotoxicity assays performed in the IPEC-J2 cell line treated with 5 µg of total protein of cell-free, bacterial lysates of either *E. faecium* SF68 (blue bar) or *E. avium* IMT39925 (red bar) for 24 h prior to the assays. Controls included untreated cells (open bar) or cells treated with lysis buffer to determine the maximum LDH release (LDH-max). The results shown are the averages of at least three, independent assays.

To exclude the possibility that the apparent inhibitory effects of the cell-free, bacterial lysates on NF-κB activation was a characteristic of the IPEC-J2 porcine intestinal epithelial cell line, we also constructed cell lines harboring the same NF-κB luciferase reporter in intestinal epithelial cell lines of both human (Caco-2) and murine (MODE-K) origin, and performed the same assays using cell-free lysates of both *E. faecium* SF68 and *E. avium*. As shown in Figure 4(a), although the degree of inhibition for the *E. faecium* SF68 treatments were slightly different in the different host cell backgrounds, in all three host cell lines there was a clear inhibition of NF-κB activity relative to untreated cells. In contrast, treatments with lysates of *E. avium* showed either unchanged (IPEC-J2), or higher levels of NF-κB activity (Caco-2, MODE-K). Notably, the relative degree of NF-κB activity in all three host cell backgrounds comparing the effects of *E. avium* to *E. faecium* SF68 lysates were within the same range, six- to eightfold, indicating the elevated levels of NF-κB activity in the Caco-2 and MODE-K cells lines were likely a characteristic of the cancerous or transformed nature of the two cell lines, but which were similarly inhibited by the *E. faecium* SF68 bacterial lysates. These results supported the conclusion that the inhibitory effects on NF-κB activation were neither host cell background nor NF-κB reporter-dependent. Likewise, consistent with the results shown in Figure 4(a), neither the *E. faecium* SF68 nor the *E. avium* lysate treatments showed signs of increased host cell cytotoxicity (Figure 4(b)).

### The inhibitory effect of E. faecium SF68 on NF-κB activation levels is reversible

**Figure 5.**
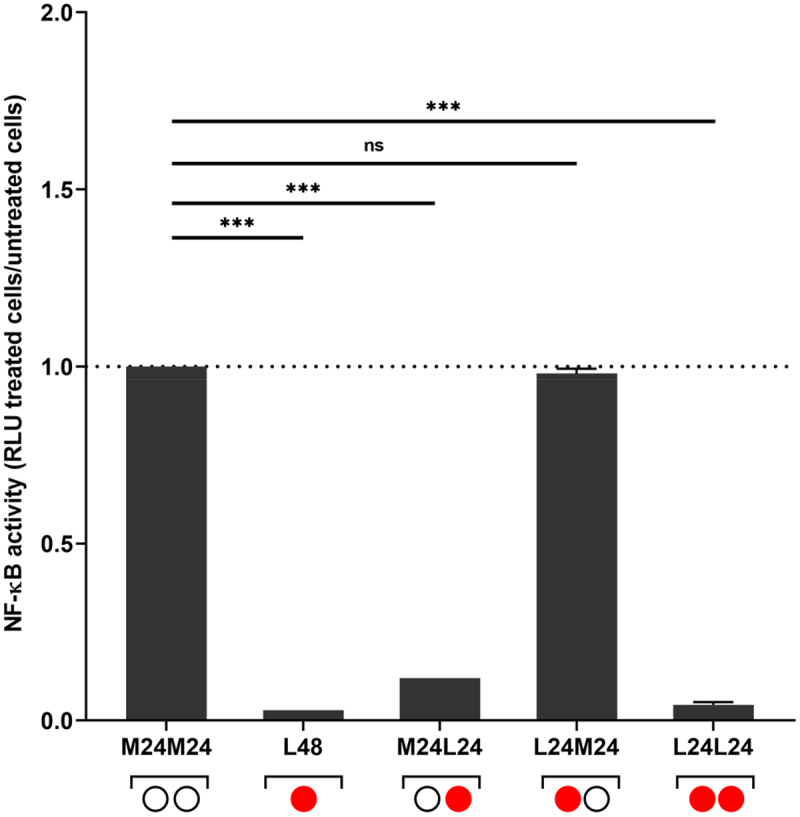
The inhibitory effect of *E. faecium* SF68 on NF-κB activation is reversible. Wells containing confluent monolayers of the IPEC-J2/K6 reporter cell line were treated with 5 µg of total protein of whole-cell, bacterial lysates of *E. faecium* SF68 (red circles) 48 h prior to determination of NF-κB activity (L24 and L48, red circles), or left untreated (M24, open circles). 24 h post-challenge, the cell culture medium was removed and replaced with either cell culture medium alone (L24/M24), or medium containing 5 µg of protein of *E. faecium* SF68 lysate (L24/L24), and incubated an additional 24 h. Controls included wells with no change of medium (L48), untreated cells (M24/M24), or cells left untreated for the first 24 h, followed by addition of 5 µg of lysate protein for the remaining 24 h (M24/L24). At 48 h, the NF-κB activity for all combinations were determined. The results shown are the relative NF-κB activities (ratio RLU treated/untreated cells) compared to untreated cells (M24/M24; dotted line). The results shown are representative of at least two, independent assays.

To determine whether the inhibitory effects of *E. faecium* SF68 lysates on NF-κB activity was reversible, cells were pre-treated with the lysates for 24 h followed by replacement of the cell culture medium with either fresh medium alone or containing bacterial lysates again for an additional 24 h. As shown in (Figure 5), replacement of the cell culture medium without lysate resulted in full recovery of NF-κB activity, whereas replacement and incubation with fresh medium containing *E. faecium* SF68 bacterial lysates continued to suppress NF-κB activity below the basal levels of activity seen in untreated, control cells. The observation that NF-κB activity fully recoverd after replacement of the media without *E. faecium* SF68 lysates further supported the conclusion that the observed NF-κB inhibition was not due to host cell killing effects, at least not for the duration of the experiments (48 h).

### Arginine deiminase (ADI) is the immuno-modulatory factor of E. faecium SF68

To determine the nature of the NF-κB inhibitory factor(s), we subjected the cell-free bacterial lysates to either proteinase K treatment or heat-treatment at 100°C for 10 min. prior to performing the NF-κB activation assays. In both cases, we observed a complete loss of inhibitory activity by the lysates, indicating that the immuno-modulatory factor was likely proteinaceous in nature (see supplementary Figure S4).

**Figure 6.**
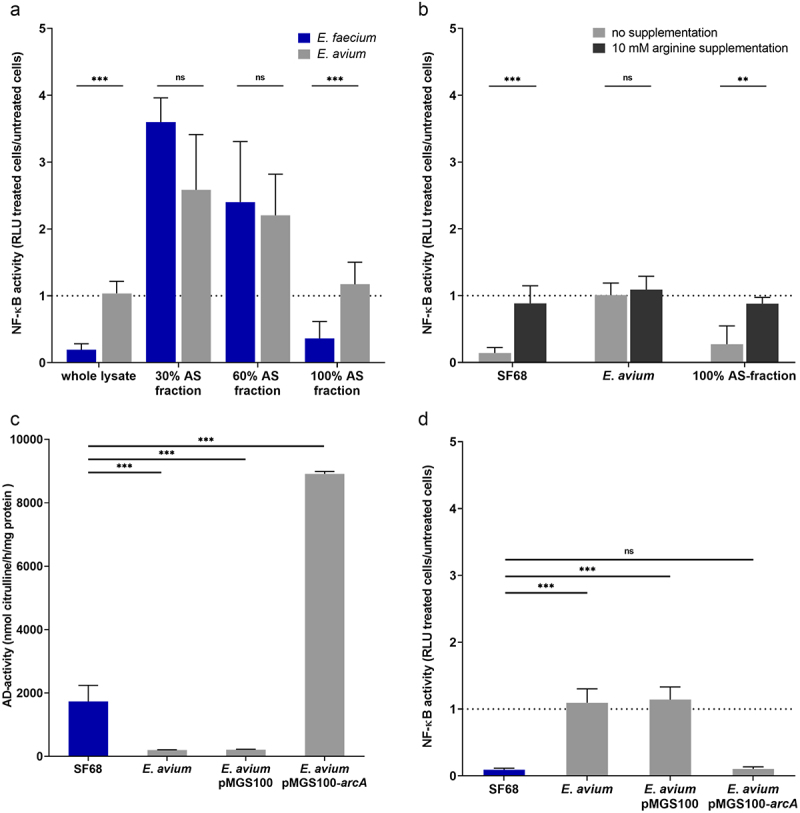
Protein fractions of *E. faecium* SF68 lysates containing arginine deiminase (ADI) inhibit NF-κB activity. (a) Total protein of cell-free, bacterial lysates or ammonium sulfate protein fractions lysates of *E. faecium* SF68 (blue bars) or *E. avium* (gray bars) were screened for effects on NF-κB activity in the IPEC-J2/K6 cell line. A total of 5 µg of total protein from either whole lysates, or the ammonium sulfate fractions as indicated in the figure, were added to confluent monolayers and incubated for 24 h prior to determination of NF-κB activity. The values shown are the relative ratios of treated cells normalized to the values for untreated, control cells (dotted line). The results shown are the averages of at least three, independent assays, and at least two, independent ammonium sulfate preparations. (b) NF-κB activity was determined for cells treated for 24 h with either cell-free, bacterial lysates of *E. faecium* SF68, *E. avium* IMT39925, or the 100% AS fractions of *E. faecium* SF68 lysates in the absence (gray bars) or presence of 10 mM L-arginine in the cell culture medium (black bars). The dotted line indicates the normalized NF-κB activity of untreated, control cells. (c) Arginine deiminase (ADI) activity determined for equivalent total protein (50 µg) of bacterial lysates of *E. faecium* SF68, *E. avium* IMT39925 (UW11197), *E. avium* harboring the vector plasmid pMGS100 (vector), or *E. avium* harboring the cloned *E. faecium* SF68 *arcA* gene in plasmid pMGS100-*arcA*_SF68_^+^ (*arcA*^+^). Assays were performed in reaction buffer containing 10 mM L-arginine for 2 h. (d) Total protein samples (5 µg) of bacterial lysates of *E. faecium* SF68, *E. avium* IMT39925, and *E. avium* harboring either the vecto*r plasmid* pMGS100 (vector) or the cloned *E. faecium* SF68 *arcA* gene in plasmid pMGS100-*arcA*_SF68_^+^ (*arcA*^+^) were used to treat IPEC-J2/K6 cells for 24 h prior to determination of NF-κB activities. Shown are the relative ratios of NF-κB activity of treated cells normalized to the values for untreated, control cells (dotted line). The results shown in all panels are the averages of at least three, independent assays.

As both heat inactivation and proteinase K digestions suggested the NF-κB inhibitory factor(s) was likely a protein, total protein of the *E. faecium* SF68 lysates were subsequently fractionated by ammonium sulfate (AS) precipitation, followed by assays of the various protein fractions for NF-κB inhibitory activities. As shown in Figure 6(a), only proteins present in the 100% saturated AS fraction of *E. faecium* SF68 showed the same inhibition of NF-κB activation as the whole-cell lysates. Ammonium sulfate fractionation of lysates of *E. avium* performed in the same manner showed no inhibitory effects for any of the AS fractions, consistent with the absence of inhibitory effects on NF-κB activation seen with whole cell-free lysates of *E. avium* (Figures 3 and 4). Furthermore, the observations with the *E. avium* AS fractions indicated that the addition of ammonium sulfate was not responsible for the reduced NF-κB activity seen for the *E. faecium* SF68 AS fractions (Figure 6(a)). Interestingly, both the 30% and 60% *E. faecium* SF68 AS fractions had a stimulatory effect on NF-κB activity in these assays, but this effect was not significantly different from fractions of the *E. avium* whole-cell lysates.

In an effort to identify proteins specific for *E. faecium* SF68, we compared the protein banding patterns of the 100% AS fractions of *E. faecium* SF68 and *E. avium* after separation on denaturing gels. A total of 12 prominent proteins appeared to be present in the 100% AS fraction of *E. faecium* SF68 but which were not present in *E. avium* 100% AS fractions (data not shown). Samples of these proteins were excised from SDS-PAGE gels and subjected to protein identification by MALDI-TOF. Interestingly, a number of the proteins identified in the active fraction of *E. faecium* SF68 lysates which were not present in *E. avium* are involved in arginine metabolism, including arginine deiminase and ornithine carbamoyltransferase. These results were of particular interest as arginine deiminase, and arginine catabolism in general, has previously been shown to play a role in the inhibition of human peripheral blood mononuclear cell proliferation and in the virulence of *Streptococcus pyogenes*,^38–40^ and arginine deiminase of *E. faecium* GR7 has been found to inhibit the proliferation of various cancer-derived cell lines.^41^ Indeed, arginine deprivation of cancer cells by recombinant and modified arginine deiminase of *Mycoplasma arginini* has long been considered as a potential means of inhibiting cancer cell growth and proliferation.^42,43^

### Arginine deiminase is responsible for NF-kB inhibition by E. faecium SF68 lysates

To determine whether arginine catabolism and subsequent arginine depletion of the cell culture medium was responsible for the inhibitory effects on NF-κB activation levels, we performed the NF-κB assays with the *E. faecium* SF68 lysates, the 100% AS-fractions, or *E. avium* lysates in the presence or absence of excess arginine in the cell culture medium. As seen in Figure 6(b), excess arginine abolished the inhibition of NF-κB activation of cells treated with both the *E. faecium* SF68 lysates and the 100% AS fractions. Control treatments using *E. avium* lysates showed no significant changes in NF-κB activity, indicating that arginine supplementation alone did not have an intrinsic stimulatory effect on NF-κB activity that might have obscured the inhibition due to the *E. faecium* SF68 lysate treatment. These results suggested the active factor present in the 100% AS fractions was indeed the arginine deiminase.

Repeated attempts to inactivate the *arcA* gene encoding arginine deiminase in *E. faecium* SF68 were unsuccessful, owing in part to an endogenous erythromycin resistance that prevented the use of a number of common streptococcal suicide vectors. In order to verify the role of the *E. faecium* SF68 arginine deiminase in NF-κB inhibition, the *arcA* gene, encoding arginine deiminase (ADI) of *E. faecium* SF68, was therefore cloned into the *E. coli-Enterococcus* shuttle vector pMGS100, and introduced into *E. avium* by electroporation. As shown in Figure 6(c, e). *avium* harboring the pMGS100 plasmid vector showed no ADI activity, whereas *E. avium* harboring the cloned *arcA* gene of *E. faecium* SF68 exhibited high, constitutive ADI activity. Furthermore, as shown in Figure 6(d), cell-free lysates of the transformed *E. avium* isolate were also found to inhibit NF-κB activation in IPEC-J2/K6 cells to the same degree as *E. faecium* SF68 lysates. In contrast, lysates derived from *E. avium* harboring the empty pMGS100 vector showed no inhibitory effects on NF-κB activation.

These results indicated that the inhibition of NF-κB activity by *E. faecium* SF68 and other *Enterococcus* strains and isolates was likely due to ADI expression. To determine whether there was a correlation between arginine deiminase activity and NF-κB inhibition, we screened all enterococcal isolates that showed inhibition of NF-κB activity, both commensal and clinical isolates, for ADI activity. All isolates that showed NF-κB inhibition also tested positive for ADI activity. In contrast, enterococcal isolates with no inhibitory effects on NF-κB activity were also found to be negative for ADI activity, except for isolates of *E. gallinarum* and *E. casseliflavus*, which were positive for ADI activity (supplementary Table S2). Notably, *E. gallinarum* and *E. casseliflavus* are exceptional among the Enterococci, as both are flagellated and motile.^44^ As *E. gallinarum* flagellin is a strong stimulator of NF-κB activation,^45^ the apparent lack of NF-κB inhibition was likely due to the presence of flagella in these isolates, which could lead to rapid, high levels of activation, masking the inhibition observed in our standard 24 h assays. These results therefore indicated that *E. faecium* SF68 arginine deiminase was most likely responsible for the observed NF-κB inhibition in both the cell-free bacterial lysates, and the 100% AS fractions of *E. faecium* SF68 containing ADI. In other experiments, we also verified that gentamicin-killed, intact *E. faecium* SF68 which also showed NF-κB inhibition (Figure 1(a)) also retained ADI activity (data not shown).

### E. faecium SF68 inhibits NF-*_k_*B activation by TLR and NOD ligands

**Figure 7.**
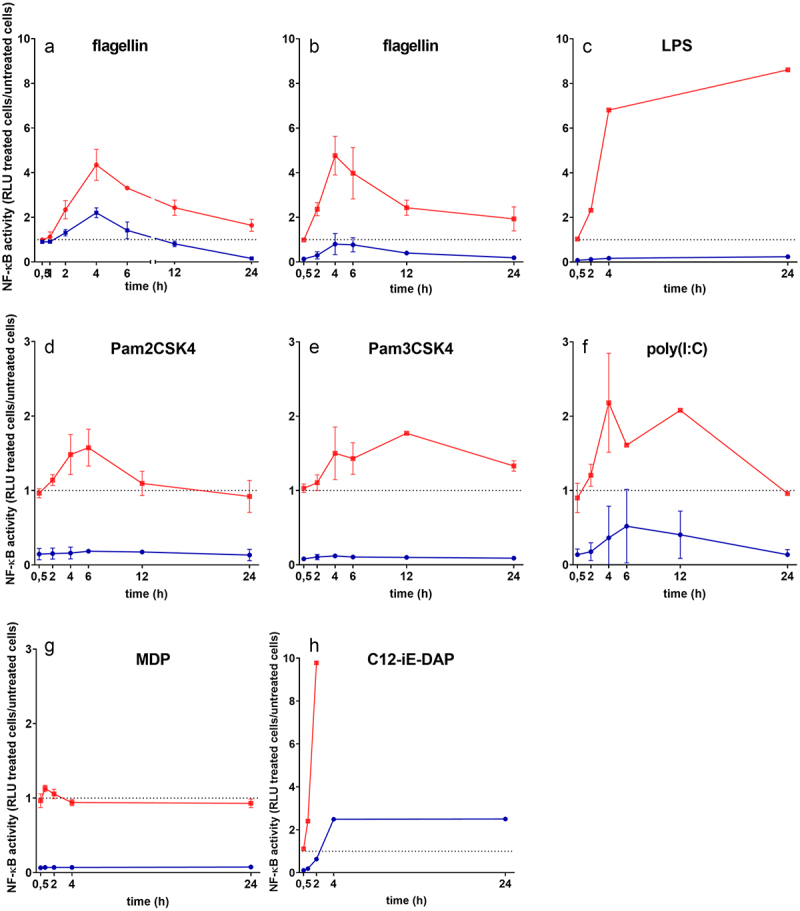
*E. faecium* SF68 inhibits host cell responses to TLR and NOD protein ligands. (a) Confluent monolayers of the IPEC-J2/K6 reporter cell line were treated with either 5 µg of total protein from bacterial lysates of *E. faecium* SF68 (blue line) or purified *Salmonella* flagellin (1.5 µg), and at the times indicated, replicate wells were sampled for NF-κB activity starting at 30 min. post-challenge. In panels B-H, cells were pre-treated for 24 h with 5 µg of *E. faecium* SF68 cell-free lysate (blue line) or left untreated (red line) prior to addition of the indicated TLR or NOD agonists, and NF-κB activity was determined for replicate wells at the times indicated in the figures. (b) TLR5 ligand, flagellin (1.5 µg). (c) TLR4 ligand, purified *E. coli* LPS (50 µg). (d) TLR2 ligand, Pam2CSK4 (1 µg). (e) TLR1/TLR2 ligand, Pam3CSK4 (1 µg). (f) 10 µg of the TLR3 ligand, poly(I:C). (g) 10 µg of the NOD2 ligand, muramyl dipeptide, (MDP). (h) 50 µg of the NOD1 ligand, acylated iE-DAP (C12-iE-DAP). In all panels, the dotted line indicates the normalized NF-κB activity of untreated, control cells. The results shown in all panels are the averages of at least two, independent assays.

A challenge of host cells with total bacterial lysates would be expected to result in activation of intestinal epithelial innate immune responses through the NF-κB signaling pathway *via* Toll-like receptor (TLR) or other receptor pathways.^45–48^ While the earliest time point (4 h post-treatment) in Figure 2(a) suggested the host cells were capable of responding with increased NF-κB activation for a short time post-challenge with the *E. faecium* lysates, we were interested to know how treatment of cells with *E. faecium* SF68 would compare to NF-κB activation in response to other, non-*E. faecium* related ligands. As seen in Figure 7(a), when challenged with *E. faecium* SF68 lysates, cells responded with a peak in NF-κB activation within the first 4 h post-challenge. However, rather than returning to either the basal level or a new, elevated activation level, NF-κB activity continued to decline over the entire 24 h period of the assay to around only 10% of the basal, pre-challenge level of activation seen in untreated cells, consistent with the results shown in Figure 2(a). Whereas a decline in NF-κB activity following an initial stimulation would be expected due to the NF-κB-dependent expression of its own inhibitors,^49,50^ the reduction in activity to levels below the basal, maintenance activity was unlikely to be a normal response. NF-κB is known to regulate the expression of a large number of genes involved in host cell growth, metabolism, and proliferation in addition to its role as a central regulator of immune and inflammatory responses.^23,29^ The regulation of metabolic, house-keeping gene expression explains the low, but non-zero levels of activation seen in even resting cells.^51^

To determine whether these results were a result of the treatment or a characteristic of the IPEC-J2/K6 reporter cell line, we performed the same experiment using the TLR5 ligand, flagellin. As seen in Figure 7(a), there was a rapid activation of NF-κB at early times post-challenge, with a peak around 4 h post-challenge, similar to the kinetics observed for the *E. faecium* SF68 cell lysates. However, the kinetics of the decline phase of NF-κB activation were very different, remaining at all times above the pre-challenge basal level, with an apparent slow decline toward the basal level of NF-κB activity present in untreated, resting cells. Similar results for flagellin were observed for incubation times of up to 48 h (data not shown), suggesting that the lower levels of NF-κB activity seen in *E. faecium* SF68 treated cells was not simply a matter of the initial magnitude of activation. The final levels of NF-κB activity were below that of both untreated cells and at late times post-challenge of TLR ligand-activated cells, both of which showed a plateau representing NF-κB activity consistent with levels required for growth, proliferation, and host cell maintenance. This is also supported by the NF-κB activation kinetics for the TLR2 ligand, Pam2CSK4, which showed a comparable magnitude of NF-κB activation 4 h to 6 h post-challenge as the *E. faecium* SF68 treatment (compare Figure 7 (a) and (d)), yet the TLR2-mediated activation declined to normal, basal levels by 24 h post-challenge, whereas the *E. faecium* SF68 treated cells showed only 10% the basal NF-κB activity at the same time post-challenge. These results indicated that the severe inhibition of NF-κB activity was a phenomenon beyond the normal, self-regulatory, down-regulation of NF-κB on its own activity. Furthermore, the results indicated this effect was a characteristic of the *E. faecium* SF68 lysates, and not a peculiarity of either the host cell line or the NF-κB reporter fusion.

To determine whether the inhibitory effects of *E. faecium* SF68 on NF-κB activation would interfere with host cell responses to other immunostimulatory ligands, we also performed challenge studies with host cells pre-treated with lysates followed 24 h later by a challenge with different TLR- and NOD protein-ligands. As shown in Figure 7(b), pre-treatment of the host cells with *E. faecium* SF68 severely reduced the ability of host cells to respond with NF-κB activation to flagellin, the TLR5 ligand. While there appeared to be a low-level capacity for NF-κB activation in such pre-treated cells, the peak of NF-κB activation did not rise above the basal level of activity of untreated cells. Similar results were observed for the TLR4, TLR2/TLR6, and TLR1/TLR2 ligands LPS, Pam2CSK4, and Pam3CSK4, respectively (Figure 7(c–e)). Interestingly, poly(I:C), a TLR3 ligand, also showed a severely reduced NF-κB activation response in cells pre-treated with *E. faecium* SF68 (figure 7(f)). Cells pre-treated with *E. faecium* SF68 cell-free lysates showed no response to the NOD2 ligand, muramyldipeptide (MurNAc-L-Ala-D-isoGln, MDP); however, the IPEC-J2/K6 cell line also showed only a minimal response to MDP alone (Figure 7(g)). In contrast, the IPEC-J2/K6 cell line responded strongly to a challenge with the NOD1 agonist, acylated γ-D-glutamyl-*meso*-DAP (C12-iE-DAP), and cells pre-treated wth *E. faecium* SF68 remained partially capable of NF-κB activation in response to C12-iE-DAP, although far below the levels of NF-κB activation seen for untreated cells (Figure 7(h)).

### E. faecium SF68 treatment interferes with phosphorylation of NF-κB-p65 at serine 536

The preceding results indicated that host cells challenged with *E. faecium* SF68 lysates were severely compromised in their ability to respond with activation of NF-κB to immunostimulatory signals. As noted above, the activation of NF-κB in the cytosol requires phosphorylation of the inhibitor, IκBα, to release NF-κB for translocation into the cell nucleus. However, phosphorylation is also known to be important for the regulation and selectivity of gene expression by NF-κB itself, and the RelA(p65) subunit is one of the most studied NF-κB phosphorylation targets.^28^ We were therefore interested to know the phosphorylation status of NF-κB, in particular phosphorylation at serine residue 536 (S536), located in the transactivation domain of RelA(p65), a modification known to play a role in both the turnover and activity of RelA(p65) as well as providing an alternative means of activation independent of IκBα.^28,52–54^

**Figure 8.**
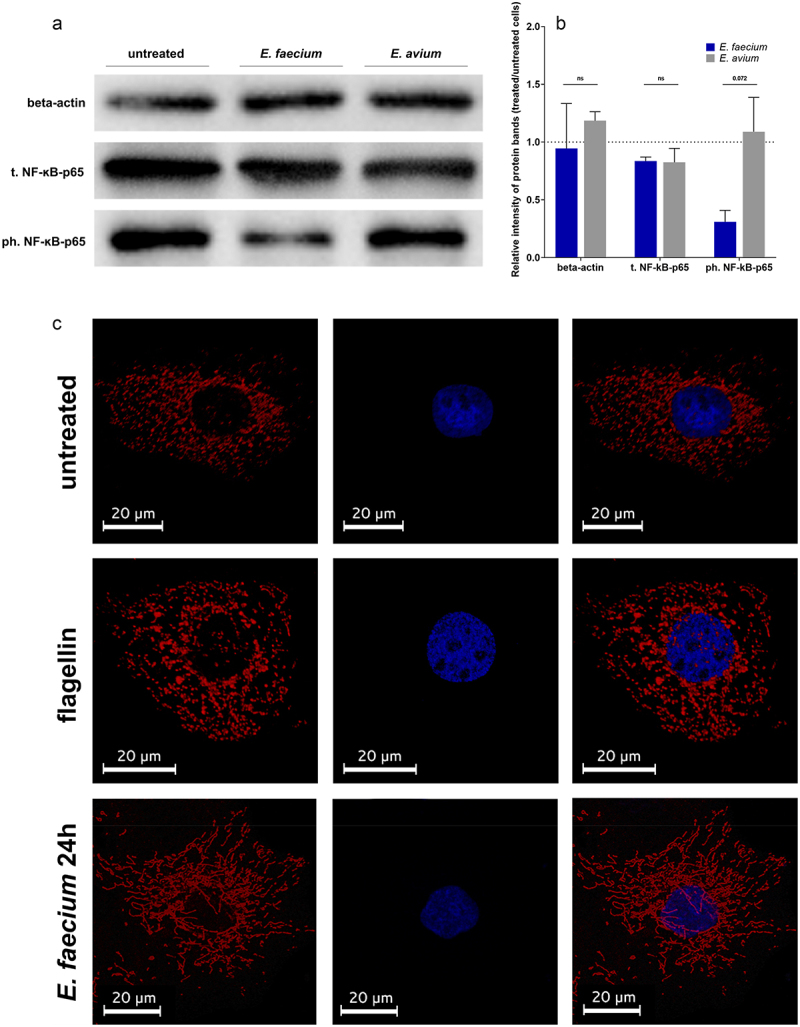
*E. faecium* SF68 inhibits NF-κB phosphorylation at Serine 536. (a) Western blot analyses of total NF-κB(p65) (t. NF-κB-p65) and its Ser536 phosphorylated form (ph.-NF-κB-p65) in IPEC-J2 cells either untreated or treated with 5 µg of total protein of cell-free lysates of either *E. faecium* SF68 or *E. avium* IMT39925 for 24 h as indicated above the blots. The results shown are representative of two, independent determinations. (b) β-actin (upper row in A, beta-actin) was used as a normalization housekeeping protein for determination of the relative levels of NF-κB(p65) and phospho-NF-κB(p65) levels in cells treated with 5 µg of total protein of cells pre-treated with either *E. faecium* SF68 (blue bars) or *E. avium* IMT39925 (gray bars). The dotted line indicates the normalized expression level of the same genes in untreated, control cells. (c) Immunofluorescence micrographs of IPEC-J2 cells after 24 h of incubation either untreated (upper row), in the presence of flagellin (1.5 µg, middle row), or 5 µg of total lysate protein of *E. faecium* SF68. After 24 h incubation, cells were fixed, and stained for total NF-κB(p65) with primary anti-NF-κB(p65) antibody and secondary Cy5-labeled antibody (red fluorescence) and DAPI for cell nuclei (blue fluorescence). The focus depth of the micrographs were chosen to allow visualization of the cell nucleus. The results shown are representative immunofluorescence micrographs from at least two, independent experiments.

A Western blot analysis of cells treated with *E. faecium* SF68 lysates (Figure 8(a)) showed similar levels of total NF-κB(p65) compared to untreated, control cells, but reduced levels of phospho-NF-κB(p65) (Figure 8(b)). In contrast, host cells treated with *E. avium* lysates contained similar total NF-κB(p65) and phospho-NF-κB(p65) levels compared to untreated cells, as expected. Immunofluorescence microscopy images of IPEC-J2 cells stained for total NF-κB(p65) indicated qualitatively higher levels of NF-κB present in the cytoplasm of cells treated with *E. faecium* SF68 lysates compared to untreated cells and cells treated with flagellin for 4 h (Figure 8(c)). Furthermore, NF-κB(p65) of cells treated with *E. faecium* SF68 lysates appeared to accumulate in the cytoplasm as aggregates, obscuring the cell nucleus. In contrast, in flagellin-treated cells, there was a clear, nuclear accumulation of NF-κB visible as puncta within the nucleus in treated cells compared to untreated cells.

## *E. faecium SF68 inhibits activation of the JNK*(*AP-1*) *pathway and inflammatory gene expression*

In addition to NF-κB, the c-Jun-N-terminal kinase (JNK) signaling pathway is also involved in host cell responses to a large variety of signals, including TLR ligands and bacterial pathogens.^55^ The JNK pathway itself is activated by phosphorylation from any of a number of different MAP kinases, including TAK1, an MAP kinase kinase (MAP3K) also involved in the signaling pathway leading to NF-κB activation. However, the downstream activation of gene expression following JNK activation is mediated by the transcription factor activator protein-1 family of proteins, AP-1.^55^ The observation that the NF-κB response to the TLR3 agonist, poly(I:C), was also inhibited (figure 7(f)) was not necessarily unexpected, as TLR3 signaling activates the JNK(AP-1) pathway in addition to NF-κB, therefore NF-κB activation may not have been expected. On the other hand, LPS is generally known to lead to NF-κB activation through TLR4 signaling, but can also activate the JNK(AP-1) pathway.^46^ It was therefore possible that the severely reduced NF-kB activation in response to the TLR4 and TLR3 agonists LPS and poly(I:C) seen in Figure 7 (c) and (d), respectively, may have nevertheless resulted in activation of the JNK(AP-1) pathway.

**Figure 9.**
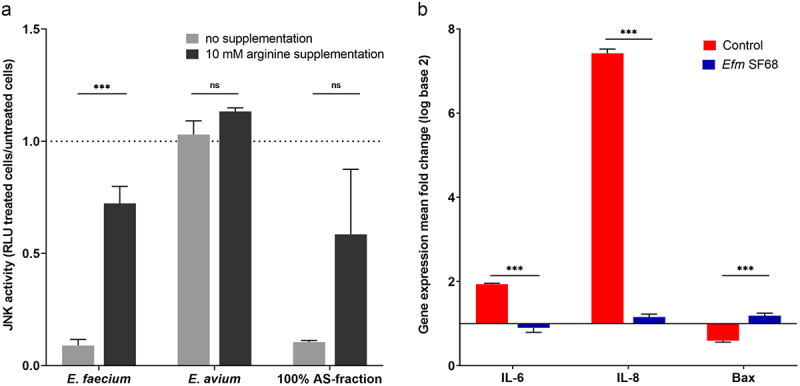
*E. faecium* SF68 inhibits the host cell JNK(AP-1) signaling pathway and gene expression responses to flagellin. (a) Confluent monolayers of the IPEC-J2/D6 cell line harboring a JNK(AP-1) luciferase reporter were treated with 5 µg of protein of either *E. faecium* SF68 or *E. avium* IMT39925 bacterial lysates, or 5 µg of total protein of the 100% AS fraction of *E. faecium* SF68 in the presence (filled bars) or absence (gray bars) of 10 mM arginine. After treatment for 24 h, the JNK(AP-1) activity was determined relative to untreated, control cells (dotted line) determined in parallel and for the same incubation times. (b) Gene expression levels for the pro-inflammatory cytokine IL-6, chemokine IL-8 (CXCL8), and apoptosis regulatory protein Bax genes in IPEC-J2 cells treated with (blue bars) or without (red bars) pre-treatment of *E. faecium* SF68 lysate for 24 h followed by a challenge with flagellin for 4 h. Differential expression was determined using the 2^−ΔΔCt^ method. The data shown are the averages of three, independent assays and are expressed as the mean fold-change relative to untreated cells.

To determine whether additional host cell signaling pathways might be affected by *E. faecium* SF68, IPEC-J2 cells harboring a JNK(AP-1) luciferase reporter fusion (IPEC-J2/D6) were treated with either *E. faecium* SF68 lysates or the 100% AS-fraction of the *E. faecium* lysates in the same manner as in the NF-κB assays. As shown in Figure 9(a), host cells treated for 24 h showed a significant reduction in JNK activation levels with the *E. faecium* SF68 cell-free lysates and clear reductions with the 100% AS-fractions, similar to the inhibition observed for NF-κB activity. In contrast, no inhibition was observed for whole-cell lysates of the *E. avium* strain. Supplementation of the cell cultures with excess arginine also restored the JNK activity in treated cells in the same manner as for NF-κB, indicating that inhibition of the JNK(AP-1) signaling pathway was also due to the ADI activity of the *E. faecium* SF68 bacterial lysates and 100% AS fractions.

To confirm that the inhibition of NF-κB activity of cells treated with *E. faecium* SF68 lysates would also interfere with downstream responses of immune-associated gene expression, we also determined the effects of pre-treatment with *E. faecium* SF68 lysates on NF-κB-dependent genes. As shown in Figure 9(b), we observed a significantly attenuated upregulation in gene expression levels in response to flagellin for the pro-inflammatory cytokine IL-6 and the neutrophil chemoattractant chemokine IL-8 genes in IPEC-J2 cells pre-treated with *E. faecium* SF68 compared to untreated cells. Notably, little or no change in gene expression was observed for the pro-apoptotic Bcl-2 homologue Bax gene, consistent with the observations shown in supplementary Figure S2C, indicating no activation of apoptosis pathways in cells treated with *E. faecium* SF68 lysates.

## Discussion

*E. faecium* SF68 is a probiotic, bacterial strain primarily used in therapeutic applications for acute diarrhea and enteritis in humans and animals.^10–13^ As noted in the introduction, we had previously observed an apparent general reduction in immune-associated gene expression in intestinal and associated lymphoid tissues in *in vivo* feeding trials with *E. faecium* SF68 in otherwise healthy, weaning piglets as well as infection challenge studies.^21^ These findings led to the suggestion that *E. faecium* SF68 has a direct anti-inflammatory or immune-suppression effect on local innate immune responses of intestinal epithelial and lymphoid tissues. Here, we show that the prior observations of immuno-modulatory effects of *E. faecium* SF68 are most likely due to expression of arginine deiminase (ADI), resulting in arginine deprivation of host cells with subsequent loss of NF-κB and JNK(AP-1) signaling pathway functions.

Arginine metabolism has become an increasingly important focus of research in the fields of cancer therapy, innate immune responses, and bacterial pathogenesis. A wide variety of human cancers are known to be auxotrophic for arginine, which explained the strong inhibition of tumor growth by the arginine deiminase (ADI) of *Mycoplasma arginini* and led to clinical trials using ADI as an anti-cancer therapy.^42,56–59^ Arginine also plays a key role as a signal regulating the activity of mTORC1 (mechanistic target of rapamycin complex 1), a central regulator of host cell metabolism and autophagy.^60–65^ The arginine pools are also involved in host cell innate immune responses where they can serve as a source of antimicrobial reactive nitrogen species. On the other hand, ADI has also been shown to play a large role in the pathogenesis of various bacterial species, supporting their survival within the host.^38–40,66–68^ Interestingly, mTORC1 is also a target for a number of bacterial pathogens as a means of immune evasion.^69–71^

In this study, we show that the arginine deiminase (ADI) of the probiotic strain *E. faecium* SF68 inhibits the activation of the NF-κB pathway in intestinal epithelial cells of human, porcine, and murine origins, as well as the JNK(AP-1) pathway in the IPEC-J2 cell line background. The inhibition results in severe reductions in innate immune signaling responses to a variety of TLR and NOD agonists, as shown in Figure 7. Previous studies have also reported effects on NF-κB, MAPK, and JNK(AP-1) signaling pathways by *Enterococcus* spp. or their products. However, the reported effects have ranged from activation to attenuation, required either contact with host cells or involved secreted bacterial products.^72–76^ Here, we show that all *E. faecium* and *E. faecalis* isolates, regardless of the source, show the same NF-κB inhibition, correlating with the presence of ADI, a part of the core genome of these and other *Enterococcus* spp. (Figures 2 and 3, and supplementary Figure S3). We suggest that a possible explanation for the often contradictory results of the *in vitro* studies is likely due to the duration of the treatments. As shown in Figures 1, 2 and 7, there is clearly an initial host cell response to *E. faecium* SF68 treatments 4 h post-treatment, as expected, but the basal activity levels of both NF-κB and JNK(AP-1) in resting cells is inhibited up to 90% by 24 h post-treatment. While a given isolate may express additional, variable cell-wall-bound or secreted products affecting host cell signaling pathways, our results indicate that all *Enterococcus* spp. capable of ADI expression will show long-term inhibition of NF-κB and JNK(AP-1) activation.

This suggestion has implications for observations from prior *in vivo* studies. As noted in the introduction, clinical trials in humans and animal studies have shown significant, anti-inflammatory effects of *E. faecium* SF68 treatments *in vivo*.^12,13^ However, in two, independent *Salmonella* challenge studies with weaned piglets, higher pathogen loads were found at systemic sites in piglets treated with *E. faecium* SF68 compared to the control groups.^19,20^ In addition, we found significantly delayed immune cell proliferative responses to both mitogen and UV-killed *Salmonella* antigens in the *E. faecium* SF68-treated animal groups,^21^ an observation consistent with an earlier report with purified *Streptococcus pyogenes* ADI and human PBMC preparations.^38^ In our previous animal study, we found significant reductions in immune-associated gene expression in intestinal tissues, mesenteric lymph nodes and spleen. Whereas reductions in pro-inflammatory gene expression (IL-8) was expected based on previous clinical studies on the anti-inflammatory effects of *E. faecium* SF68 in human and animal trials, there were also significant reductions in expression of IL-10, and T-cell co-activator CD86(B7.2) genes as well, *i.e*. reductions in both anti-inflammatory and adaptive immune response gene expression, suggesting a general suppression of immune-associated genes.^21^ As all of these genes have been shown to be NF-κB-dependent,^29^ the results of the current study suggest an explanation for the prior *in vivo* studies.

The arginine deiminase of *E. faecium* SF68 is clearly not secreted, as the ADI activity was not reproducibly apparent in bacterial culture supernatants (data not shown), although it may be surface bound as is the case in *Streptococcus pyogenes* and *S. suis*.^38,39,77^ This raises the question as to how ADI activity is able to affect host cell signaling pathways in intestinal epithelial and gut-associated lymphoid tissues *in vivo* when present as either a bacterial cytosolic or cell wall-bound protein factor. The observation that the ADI activity is present in killed, intact bacteria and cell-free, whole bacterial lysates of *E. faecium* SF68 as well as ammonium sulfate protein fractions indicates that ADI does not require active bacterial metabolism for activity. In addition, ADI from a number of bacterial species is known to be active under highly acidic conditions, which has led to it being regarded as a virulence factor, involved in bacterial survival in low pH environments, including the lysosome/phagolysosome when internalized by host cells.^38–40,66–68^ These observations suggest that *E. faecium* SF68 need not be present *in vivo* as a viable, probiotic strain in order to show the effects on host cell signaling we have demonstrated here. Internalization by host cells could well result in bacterial killing within the lysosome/phagolysosome, but the presence of cell wall-bound or intracellular ADI in killed bacteria, or release of internal ADI by lysozyme digestion within the lysosome would allow ADI to deplete the lysosome of arginine, and raise the pH of the lysosome/phagolysosome through release of ammonia, inactivating many of the hydrolytic, proteolytic, and lipolytic enzymes of the lysosome. In other words, even if the probiotic *E. faecium* SF68 is killed by the host cells, the activity or release of ADI would nevertheless lead to arginine depletion and neutralization of the acidic pH of the lysosomal compartments, the latter effect of which would eliminate an important antimicrobial defense mechanism of host cells.

In addition to neutralization of the lysosomal pH, we suggest an additional effect of internalized *E. faecium* SF68 and/or ADI which would explain the severe inhibition of NF-kB and JNK(AP-1) signaling pathways observed in this study. The observation that both signaling pathways showed severe reductions in activation was somewhat unexpected, as the pathways are largely independent of one another, although they share a number of upstream regulators, such as TAK1, involved in recruitment of IKK kinase.^23^ As noted above, mTORC1 is a central regulator of host cell metabolism, growth, and autophagy, and the activity of mTORC1 is dependent on arginine, among other signals. Furthermore, the lysosomal arginine pools play a key role in the regulation (activation) of mTORC1.^65,78,79^ An early study found that mTORC1 is involved in the regulation of NF-κB through interaction with the IKK complex which in turn is responsible for phosphorylation and initiation of degradation of the NF-κB inhibitor, IκB, explaining how arginine depletion would affect NF-κB activity.^80^ Likewise, defects in lysosome function and mTORC1 activation have been reported to be essential for the phosphorylation (activation) of JNK.^81^ More recently, innate immune signaling through TLR4, the LPS receptor, has also been found to involve mTORC1/2 and the JNK and MAPK pathways.^82^ We suggest that all of the observations from both this study and prior *in vitro* and *in vivo* studies can be explained by ADI-dependent depletion of arginine pools and inactivation of mTORC1 of host cells, with the possible qualification that it would likely be a localized effect *in vivo, i.e*. affecting intestinal epithelia and gut-associated immune cell populations, rather than systemic effects. This would be consistent with the results from our own^21^ and other studies.

Finally, the results of our study raises serious questions regarding the use of *Enterococcus* strains as probiotics for therapeutic purposes. While the use of *E. faecium* SF68 as a therapy for symptoms of intestinal inflammation has yielded encouraging results in the past,^10–13^ if one of the major mechanisms of action is a general inhibition of innate immune signaling by ADI in intestinal tissues, including immune cell populations, this would be expected to have consequences in the event of secondary bacterial infections during the treatment period, particularly for invasive pathogens. That ADI alone can inhibit the proliferation of host cells *in vivo* has been the basis for clinical trials in humans for certain forms of cancers. Our group and others have also noted deleterious effects in challenge experiments with *Salmonella* in animal studies with post-weaning piglets, where pre-treatment with *E. faecium* SF68 resulted in higher bacterial loads at systemic organ sites such as tonsils and spleen.^19–21^ We suggest the latter observations reflected a dampened, intestinal innate immune response which led to elevated rates of intestinal tissue invasion by the facultative intracellular pathogen *S*. Typhimurium and subsequent spread to systemic sites. Notably, in those studies the humoral immune response reflected in *Salmonella*-specific antibody titers showed elevations; however, the antibody titers were consistent with the higher bacterial loads at systemic sites.^19,20^ Whereas Enterococcal probiotics such as *E. faecium* SF68 clearly have applications in alleviating intestinal inflammation, we suggest that where the clinical benefits are based on what is arguably a bacterial virulence factor with such wide-ranging inhibitory effects on innate immune responses, the decision for administration of *E. faecium* SF68 and other Enterococcal probiotics for prophylactic and therapeutic purposes should be made cautiously.

## Materials and methods

### Bacterial strains, plasmids, and growth conditions

The probiotic *Enterococcus faecium* SF68 (NCIMB 10415, Cernelle SF68, Cylactin) strain was chosen as a representative, probiotic *E. faecium* strain as it is well-characterized, and has been used in a number of prior human and animal clinical trials.^10–13^ *E. faecium* SF68 was obtained directly from the proprietary company, Cerbios-Pharma SA, Lugano, Switzerland. Additional clinical and commensal strains of *E. faecium, E. avium, E. casseliflavus, E. cecorum, E. durans, E. faecalis, E. gallinarum, E. hirae*, and *E. raffinosus* are listed in supplementary Tables S1 and S2. The *E. avium* strain IMT39925 (UW11197) used as a host for the heterologous expression of the *E. faecium* SF68 arginine deiminase was provided by Dr. Guido Werner, National Reference Laboratory (NRZ) for *Staphylococcus* and *Enterococcus*, Robert Koch Institute, Wernigerode, Germany. Enterococcal isolates were grown on blood agar plates overnight at 37°C. *E. coli* strain DH5α harboring the vector pMGS100, was generously provided by Dr. Shuhei Fujimoto, Department of Microbiology, Gunma University School of Medicine, Maebashi, Japan. *E. coli* strains were grown in Luria-Bertani broth at 37°C and subcultured on Luria-Bertani agar plates. *Enterococcus* strains were routinely grown on brain heart infusion (BHI) or Todd Hewitt agar plates and liquid cultures. Where appropriate, ampicillin (100 µg/ml), kanamycin (25 µg/ml), or chloramphenicol (20 µg/ml) were added to the plates for selection. Additional references and source information regarding the bacterial strains and plasmids are found in supplementary Tables S1 and S2.

### Cell lines and cell culture conditions

The IPEC-J2 cell line is a well-characterized, non-transformed, intestinal epithelial cell line derived from jejunal epithelia of a neonatal piglet. Cells were maintained in Dulbecco’s modified Eagle medium (DMEM)/Ham’s F-12 medium (1:1) (Biochrom) supplemented with 10% inactivated fetal bovine serum (Biochrom). Cells were grown at 37°C in a humidified incubator, at 5% CO_2_. Cell lines harboring NF-κB or JNK(AP-1) chromosomal reporter fusions received 5 µg/ml puromycin (Carl Roth, Germany) as a selective agent in the medium. One day prior to experiments, the cell culture medium was removed, replaced with 1X PBS, and subsequently replaced with full medium without puromycin. The Caco-2 cell line is a human epithelial intestinal cell line, derived from a colorectal adenocarcinoma. The murine intestinal epithelial MODE-K cell line, was derived by transformation of intestinal epithelial cells of C3H/HeJ mice with the SV40 large T antigen. All cell lines were maintained under the same conditions as described for IPEC-J2 cell line. Additional references and source information regarding the cell lines are found in supplementary Table S1.

### Construction of NF-κB and JNK/AP-1 luciferase reporter cell lines

NF-κB-luciferase reporter derivatives of porcine (IPEC-J2/K6), human (Caco-2/C6), and mouse (MODE-K/H8) intestinal epithelial and IPEC-J2 MAPK/JNK(AP-1)-luciferase cell lines (IPEC-J2/D6) were constructed by infection of the cell lines with prepackaged, lentiviral vectors harboring either NF-κB- or JNK(AP-1)-luciferase reporter fusions with selection for puromycin resistance according to the manufactuerer´s instructions (Cignal Lenti Reporters, SA Biosciences, CLS-013 L and CLS-011 L, resp.). These luciferase fusion constructs encode a minimal promoter element (TATA box) preceded by a transcriptional response element specific for either NF-kB or AP-1. The luciferase is a mammalian codon-optimized, non-secreted form of the firefly luciferase gene, carrying a protein-destabilizing sequence to minimize long-term accumulation of the luciferase. After removal of dead, non-adherent cells, puromycin-resistant cells were allowed to form microcolonies, then pooled and diluted in fresh cell culture medium containing puromycin to a concentration of approximately ten cells/ml, and 100 µl of the suspension was used to seed each well of a 96-well plate. Clones derived from single cells under selection with puromycin were grown to monolayers, harvested and used to seed 25 cm^2^ flasks (Corning). All clones isolated in this manner were screened for their responses to TLR ligands and those showing the best background/induction ratios and dose responses were retained for further assays.

### Preparation of killed, intact bacteria and cell-free, bacterial lysates for co-culture assays

For preparation of killed but intact *E. faecium* SF68, the strain was grown overnight on blood or BHI agar plates at 37°C, followed by resuspension of colonies in phosphate-buffered saline (PBS) which was then adjusted to an optical density at 600 nm (OD_600_) of 1 (approximately 10^9^ CFU/ml). The bacterial suspension was then diluted 1:100 in cell culture medium containing 500 µg/ml gentamicin and incubated at 37°C for 4 h. The total killed bacterial suspensions were subsequently collected by centrifugation and concentrated by resuspension in a final total volume of 0.5 ml of cell culture medium containing 100 µg/ml gentamicin and 100 µg/ml streptomycin. The optical density was again determined and adjusted to an OD_600_ of 1. Appropriate dilutions of the bacterial suspensions were then performed to yield different multiplicities of infection (MOI) in a final volume of 0.1 ml to 96-well plates containing confluent monolayers of host cells (approximately 10^5^ cells/well). Controls included plating of different dilutions of the initial and post-gentamicin treatment suspensions to determine the input CFU/ml and efficiency of gentamicin killing.

Cell-free, whole-cell lysates of bacterial isolates were prepared using a FastPrep 24 homogenizer and 0.1 mm silica beads for lysis of Gram-positive and -negative bacteria in 2 ml tubes (Lysing Matrix B, MP Biomedicals). Approximately 10^9^/ml bacteria in 1 ml of deionized, distilled water were added to the lysis tubes, and lysed by homogenization (shaking) using a setting of 6 m/s at three bursts of 40s duration each run. The resulting lysates were sterile-filtered by passage through a 0.22 µm PVDF filter (Carl Roth, Germany) to remove debris and non-lysed bacteria. Controls for the efficiency of lysis was generally around 70–80%, indicating the lysates represented an average total protein content of 7.5 × 10^8^ CFU/lysate. The total protein concentration of the filtered lysates was determined using a bicinchoninic acid (BCA) assay, with BSA determinations in parallel for generation of standard curves, as per the manufacturer´s instructions (Thermo Scientific). The total protein concentration of these lysates, and the average total protein concentration of the lysates was between 100 and 150 μg, corresponding to total protein concentrations equivalent to that of about 6 × 10^6^ bacteria/μg. As indicated in the Figure legends, 5 μg of total protein of the cell-free lysates were used in the cell culture assays, corresponding to approximately 3 × 10^7^ CFU equivalents of total bacterial protein.

### NF-_k_B and JNK(AP-1) activation assays

The NF-κB- and JNK(AP-1)-luciferase reporter assays were performed with cells seeded onto white, flat-bottom, 96-well plates (Corning), grown to near confluency (approximately 3.2 × 10^4^ cells per well) at 37°C, and 5% CO_2_. The standard assays consisted of addition of 5 µg of total protein of the cell-free, bacterial lysates to replicate wells, and further incubation of the cells for the times indicated in the figures. Additional wells with no additions served as background (negative) controls. In co-incubation studies for host cell responses to different TLR- and NOD protein-ligands, cells were first pre-treated by addition of 5 µg of *E. faecium* SF68 cell-free lysate for 24 h, followed by addition of the TLR-ligands Pam2CSK4, Pam3CSK4, LPS, Poly (I:C) and flagellin (InvivoGen) at final concentrations of 500 ng/ml, 500 ng/ml, 5 µg/ml, 1 µg/ml and 100–150 ng/ml, respectively, unless otherwise indicated. The NOD protein ligands MDP and C12-iE-DAP (InvivoGen) were used at concentrations of 1 µg/ml and 5 µg/ml, respectively. At the times indicated in the figures, the luciferase activity was determined by addition of Bright-Glo™ Luciferase Assay System reagents (Promega) and luminescence was determined using a Synergy HT microplate reader (BioTek). In kinetics experiments, after the luciferase determinations, the plates were placed in the cell culture incubator and further incubated until the next time-point determinations.

Where indicated, cell culture medium was supplemented with an additional 10 mM L-arginine (standard concentration of arginine in DMEM:Ham´s F12 is approx. 850 µM). In other assays, the bacterial lysates of *E. faecium* SF68 strain were inactivated by either pre-heating the bacterial lysate for 10 min at 90°C or pre-treating the lysates with 1.25 µg of proteinase K (Qiagen) at 37°C for 60 min. prior to addition to the wells of growing cells. At the end of the experiment, the NF-κB or JNK(AP-1) activities were determined by addition of the Bright-Glo™ Luciferase reagent and luciferase activities determined as above (see supplementary Figure S4).

### Cytotoxicity and viability assays

Determination of the effects of cell-free, bacterial lysates and recombinant arginine deiminase on cell cytotoxicity was performed using CytoTox-ONE^TM^ Homogeneous Membrane Integrity assays, performed according to the manufacturer’s instructions (Promega). Where indicated, catalase (C1345, Sigma-Aldrich) was included at 2500 U. The cell permeable, pan-caspase inhibitor carbobenzoxy-valyl-alanyl-aspartyl-(O-methyl)-fluoromethylketone in DMSO (Z-VAD-FMK; InvivoGen) was included at a concentration of 10 µg/ml, with DMSO at the same final concentration serving as a control.

### Heterologous expression of E. faecium SF68 arginine deiminase in E. avium

The *arcA* gene of *E. faecium* SF68 was amplified by PCR from chromosomal DNA using primers containing restriction sites (underlined) for *Eag*I, primer arcAEagI (5´-TTTTTCGGCCGAACATGGATAAACCTATTCACGTTTTC-3´) and *Nru*I, primer arcANruI (5´- ATTTTTCGCGAGAGGAAATCCTGACGACAGC-3´). The PCR product was digested with *Eag*I and *Nru*I (Promega) and ligated with plasmid pMGS100 also digested with *Eag*I and *Nru*I. The resulting ligation reactions were used to transform electrocompetent *E. coli* K-12 strain DH5α by electroporation, with selection for chloramphenicol resistance. Plasmid preparations of putative clones were performed using QIAprep Spin Miniprep kits (Qiagen), and positive clones were verified by PCR and sequencing using the primers Bapro-for-01 (5´-AAAATAGTCGACTGATTGAAACTCAAGAT-3´) and NruI-seq-rev-01 (5´-GCAACGCGGGCATCCCGAT-3´). The resulting plasmid, (pMGS100-*arcA*_SF68_), harboring the *E. faecium* SF68 *arcA* gene under transcriptional control of the constitutive *E. faecalis* bacteriocin 21 *bacA* gene promoter, and the vector plasmid, pMGS100, were introduced into *E. avium* by electroporation with selecton for chloramphnicol resistance. Transformants were verified by PCR amplification and re-sequencing of putative pMGS100-*arcA*_SF68_ clones, as above, and screening for arginine deiminase (ADI) activity. For additional information regarding the electroporation of *E. avium*, and ADI assays, see supplementary Methods.

### Ammonium sulfate protein fractionation

Ammonium sulfate precipitation/fractionation of *E. faecium* SF68 and *E. avium* bacterial lysates were performed with strains grown in duplicate, 300 ml BHI broth cultures at 37°C with aeration, using standard protocols. Bacteria were collected and concentrated in two centrifugations with resuspension both times in 1X phosphate buffered saline (PBS). The concentrated bacterial suspensions were lysed using a French press at 18000 psi, with five passages. The resulting bacterial cell lysates were then cleared by centrifugation for 30 min at 4°C, 11000 x *g*. The supernatants were collected, and 10 ml was subjected to ammonium sulfate (AS) fractionation in steps of 30%, 60% and 100% (w/v) ammonium sulfate solutions by addition of the appropriate amounts of solid ammonium sulfate with stirring on ice for approximately 30 min., followed by an additional 90 min. on ice. Precipitated proteins from each fractionation step were collected by centrifugation for 30 min., at 4°C, at 11000 x *g*, and resuspended in 1X PBS. Protein concentrations of the initial French press lysates and subsequent supernatant and pellet resuspensions were performed using Micro BCA assays (Interchim). Initial characterization of the AS fractions was performed by screening a total of 5 µg of total protein present in the 30%, 60% and 100% AS fractions of *E. faecium* or *E. avium* in luminescence assays after treatment of the IPEC-J2/K6 NF-κB-luciferase reporter cell line for 24 h, as described above. For additional detailed protocols and references, see supplementary Methods.

## *Determination of arginine deiminase* (*ADI*) *activity*

Arginine deiminase (ADI) activities in the AS fractions of *E. faecium* SF68, *E. avium*, and *E. avium* transformants harboring plasmids pMGS100 or pMGS100-*arcA*_SF68_, were determined from 1:5 dilutions of protein preparations in water in a total of 100 µl added to 400 µl of 0.1 M potassium phosphate buffer, pH 6.5, and 10 mM L-arginine, and the reactions were incubated at 37°C for 2 h. At the end of the incubation, the reactions were terminated by addition of 250 µl of a sulfuric acid/orthophosphoric acid stop solution and 31.3 µL of 3% diacetyl monoxime, and the samples were boiled for 15 min. at 100°C in the dark. Reactions were allowed to cool to room temperature in the dark for 10 min. and the absorption at 440 nm was determined. Reactions without addition of bacterial lysate or AS fractions served as negative (background) controls. The ADI activity of the samples was determined from a standard curve of 0 to 100 µg of citrulline performed in parallel. The final ADI activity was calculated as the as nmol citrulline/h/mg protein. For addtional details of the ADI enzymatic assays and references, see the supplementary Methods.

Screening for ADI activity of additional *Enterococcus* isolates was also determined using arginine dihydrolase (ADI or ADH) tablets (Rosco Diagnostica, Taastrup, Denmark). Bacterial suspensions were adjusted to 4.0 McFarland in 250 µl of 0.85% NaCl solution. An ADH diagnostic tablet was added to the suspension, and 3 drops of sterile paraffin oil were overlayed to provide anaerobic conditions. The tubes were incubated at 37°C and the results were recorded at 4 h and 24 h after incubation. Positive results are indicated by a strong red color resulting from ammonia production and an alkalinization of the medium in the presence of a pH indicator, methyl red. Negative results showed either a yellow to light orange color change.

### MALDI-TOF identification of ammonium sulfate fraction proteins

Proteins present in the different AS fractions of *E. faecium* and *E. avium* lysates were separated using sodium dodecyl sulfate polyacrylamide gel electrophoresis (SDS-PAGE) followed by silver staining. The protein bands present in the active, 100% ammonium sulfate (AS) fraction of *E. faecium* SF68 lysate, which were not present in other fractions of the *E. faecium* SF68 lysate or the same AS fraction of *E. avium* lysates, were excised from the gel, destained and digested with sequencing grade trypsin at 100 µg/ml (Promega). Digested peptides were spotted onto a ground steel MTP 384 MALDI target plate (Bruker Daltonics, Germany), using the dried-droplet technique and α-Cyano-4-hydroxycinnamicacid (HCCA) (Sigma-Aldrich, Germany) matrix. Protein identification was carried out using matrix-assisted laser desorption ionization with a time-of-flight mass spectrometer (MALDI-TOF MS) (Ultraflex II TOF/TOF, Bruker Daltonics). For additional information and references regarding protein preparation and analysis for MALDI-TOF, see the supplementary Methods.

### Western blot analysis

Confluent monolayers of IPEC-J2 cells were treated with either *E. faecium* SF68 cell-free lysates, flagellin at 100 ng/ml, or cell culture medium only (untreated) for 24 h. Cells were washed with ice-cold PBS and directly lysed with Laemmli sodium dodecyl sulfate sample buffer and 1% phosphatase inhibitor cocktail 3 (Sigma-Aldrich, USA). Western blotting was performed using standard protocols. NF-κB p65 antibody was used at a dilution of 1:2000 (10745-1-AP, Proteintech Group), p-NF-κB p65 antibody (27.Ser536) at 1:300 (sc-136548, Santa Cruz Biotechnology), β-actin antibody at 1:5000 (66009-1-Ig, Proteintech Group). Secondary antibodies included goat anti-mouse IgG coupled to horseradish peroxidase (HRP) at 1:2000 (ab97040, Abcam) and goat anti-rabbit IgG (HRP) antibody at 1:1000 (A0545, Sigma-Aldrich). Quantification of the images was performed using ECL-visualizing kit (GE Healthcare Life Sciences) and Image Lab Touch software.

## *Immunofluorescence* (*IF*) *staining*

Immunofluorescent staining for the proliferation marker Ki67 was performed with IPEC-J2 cells seeded onto 12 mm glass cover slips (Carl Roth) and grown to semi-confluency, and either treated with 5 µg of total protein of *E. faecium* SF68 cell-free, bacterial lysates, or left untreated. The following day, cells were washed with 1X PBS, and fixed with ice-cold methanol for 10 min., followed by a wash with cold 1X PBS. Cells were permeabilized with 1% digitonin in PBS for 10 min., followed by two to three washes with cold, 1X PBS. The fixed cells were then incubated with mouse, monoclonal IgG1κ anti-human Ki67 antibodies (M7240, Dako Omnis) in 1% BSA overnight at 4°C. The following day, the cells were washed three times with 1X PBS, and incubated with AlexaFluor-568 labeled, goat anti-mouse IgG secondary antibodies (A11004, Invitrogen) diluted 1:1000 in 1% BSA for 30 min. at room temperature in the dark. The cover slips were washed again, twice with 1X PBS, and visualized using a Leica TCS SP-2 confocal laser scanning microscope.

For immunofluorescent staining of NF-κB, IPEC-J2 cells were treated with either 5 µg of total protein of cell-free lysates of *E. faecium* SF68 or 100 ng/ml of flagellin for 4 and 24 h. At the end of incubation, cells were fixed with 4% PFA (Carl Roth), and permeabilized for 10 min at RT with 0.1% Triton X-100 (Carl Roth). Permeabilized cells were blocked with 5% donkey serum in PBS for 1 h at room temperature. Immunostaining was conducted overnight using primary NF-κB p65 antibody at 1:300 (10745-1-AP; Proteintech) followed by a 1 h incubation with a 1:50 dilution of MFP-DY-490-Phalloidin (MFP-D490-33; MoBiTec) to label actin, and a 1:1000 dilution of donkey, anti-rabbit antibodies conjugated with the fluorophore Cy5 (711–175-152, Jackson ImmunoResearch). Cover slides were incubated with DAPI (H-1200, Vector Laboratories) for 5 min and mounted with mounting medium (P36961, Thermo Fisher) and visualized using a Leica SP8 confocal laser scanning microscope.

### Quantitative real-time PCR

Cells were seeded onto 6-well plates and grown to a confluency of approximately 90% and treated with *E. faecium* SF68 lysates or cell culture medium for 24 h followed by a 4 h treatment with flagellin at 100 ng/ml. Cells treated with only *E. faecium* SF68 lysates or cell culture medium (untreated) served as controls. RNA extraction was performed with a combination of TRIzol (Ambion, USA) and RNeasy Plus Mini Kit (Qiagen) standard extraction protocols and RNase-free DNase treatment (Promega) according to the manufacturer’s recommendations. RNA concentrations and purity were determined using NanoDrop Spectrophotemeter (Thermo Fisher) with the criteria of A_260_/A_280_ ≥ 1.9, A_260_/A_230_ ≥ 1.9 and by gel electrophoresis for the absence of RNA degradation. Complementary DNA (cDNA) was synthetized using 5 µg of purified total RNA, Revert Aid reverse transcriptase and Oligo(dT)18 primers according to the manufacturer’s instructions (Thermo Fisher).

Real-time PCRs for cytokine/chemokine (IL-6, IL-8), and apoptosis regulatory (Bax) gene expression were performed using SYBR Green SensiFAST Probe Lo-ROX Master Mix (Bioline, UK) and a StepOnePlus^TM^ Real-time PCR System (Applied Biosystems). 20 µL of final volume including 2 µL of cDNA (1:10 dilution) template was added for each sample to a MicroAmp^TM^ 96-Well Reaction Plate (Thermo Fisher) and amplified in duplicate using gene-specific primer pairs for porcine β-actin, IL-6, IL-8, and Bax. Results were normalized to the housekeeping gene for β-actin. The relative changes in gene expression was determined using the 2^−ΔΔCt^ method, with relative gene expression indicated as -fold change. For additional information and oligonucleotide sequences, see supplementary Methods and supplementary Table S3.

### Statistical analyses

Statistical analyses were performed using the SPSS software, version 25.0 (IBM). The normal distribution of data was evaluated by a 1-sample Kolmogorov–Smirnov test. Significance between the two groups were calculated by an independent, unpaired Student´s t-test. P values of ≤ 0.05 were considered statistically significant (95% confidence intervals). In the figures, statistical significance is indicated as: n.s., P > .05; *, P ≤ .05; **, P ≤ .01; ***, P ≤ .001.

## Supplementary Material

Supplemental Material

## Data Availability

Supplementary data supporting the findings of this study are openly available at https://figshare.com/ at the doi address listed below. Supplementary Figures: http://doi.org/10.6084/m9.figshare.20115950 Supplementary Methods: http://doi.org/10.6084/m9.figshare.20115902 Supplementary Table S1. Strains and Plasmids. http://doi.org/10.6084/m9.figshare.20115908 Supplementary Table S2. Enterococcus spp. http://doi.org/10.6084/m9.figshare.20115905 Supplementary Table S3. Oligonucleotides http://doi.org/10.6084/m9.figshare.20115911

## References

[1] Piddock LJ. The crisis of no new antibiotics - what is the way forward? Lancet Infect Dis. 2012;12:249–25. doi:10.1016/S1473-3099(11)70316-4 22101066

[2] Gareau MG, Sherman PM, Walker WA. Probiotics and the gut microbiota in intestinal health and disease. Nat Rev Gastroenterol Hepatol. 2010;7:503–514. Crossref. doi:10.1038/nrgastro.2010.117 20664519 PMC4748966

[3] Sanders ME, Merenstein DJ, Reid G, Gibson GR, Rastall RA. Probiotics and prebiotics in intestinal health and disease: from biology to the clinic. Nat Rev Gastroenterol Hepatol. 2019;16(10):605–616. 31296969 10.1038/s41575-019-0173-3

[4] Pagnini C, Saeed R, Bamias G, Arseneau KO, Pizarro TT, Cominelli F. Probiotics promote gut health through stimulation of epithelial innate immunity. PNAS. 2010;107(1):454–459. doi:10.1073/pnas.0910307107 20018654 PMC2806692

[5] Markowiak P, Śliżewska K. The role of probiotics, prebiotics and synbiotics in animal nutrition. Gut Pathog. 2018;10:21. 29930711 10.1186/s13099-018-0250-0 PMC5989473

[6] Sánchez B, Delgado S, Blanco-Míguez A, Lourenço A, Gueimonde M, Margolles A. Probiotics, gut microbiota, and their influence on host health and disease. Mol Nutr Food Res. 2017;61:1600240. doi:10.1002/mnfr.201600240 27500859

[7] Kim S-K, Guevarra RB, Kim Y-T, Kwon J, Kim H, Cho JH, Kim HB, Lee J-H. Role of probiotics in human gut microbiome-associated diseases. J Microbiol Biotechnol. 2019;29:1335–1340. 31434172 10.4014/jmb.1906.06064

[8] Pessione E. Lactic acid bacteria contribution to gut microbiota complexity: lights and shadows. Front Cell Infect Microbiol. 2012;2:86. doi:10.3389/fcimb.2012.00086 22919677 PMC3417654

[9] Hatti-Kaul R, Chen L, Dishisha T, El Enshasy H. Lactic acid bacteria: from starter cultures to producers of chemicals. FEMS Microbiol Lett. 2018:365. doi:10.1093/femsle/fny213 30169778

[10] Lewenstein A, Frigerio G, Moroni M. Biological properties of SF68, a new approach for the treatment of diarrheal diseases. Curr Therapeut Res. 1979;26:967–981.

[11] Wunderlich PF, Braun L, Fumagalli I, D’Apuzzo V, Heim F, Karly M, Lodi R, Politta G, Vonbank F, Zeltner L. Double-blind report on the efficacy of lactic acid-producing *Enterococcus* SF68 in the prevention of antibiotic-associated diarrhoea and in the treatment of acute diarrhoea. J Int Med Res. 1989;17:333–338. doi:10.1177/030006058901700405 2676650

[12] Franz CM, Huch M, Abriouel H, Holzapfel W, Galvez A. Enterococci as probiotics and their implications in food safety. Int J Food Microbiol. 2011;151:125–140. doi:10.1016/j.ijfoodmicro.2011.08.014 21962867

[13] Holzapfel W, Arini A, Aeschbacher M, Coppolecchia R, Pot B. *Enterococcus faecium* SF68 as a model for efficacy and safety evaluation of pharmaceutical probiotics. Benef Microbes. 2018;9:375–388. doi:10.3920/BM2017.0148 29633645

[14] Ramsey M, Hartke A, Huycke M. The physiology and metabolism of enterococci. In: Gilmore MS, Clewell DB, Ike Y, Shankar N, editors. Enterococci: from commensals to leading causes of drug resistant infection. Boston (MA):Massachusetts Eye and Ear Infirmary; 2014. p. 581–635.

[15] Marquis RE, Bender GR, Murray DR, Wong A. Arginine deiminase system and bacterial adaptation to acid environments. Appl Environ Microbiol. 1987;53:198–200. doi:10.1128/aem.53.1.198-200.1987 3103530 PMC203628

[16] Scharek L, Guth J, Reiter K, Weyrauch KD, Taras D, Schwerk P, Schierack P, Schmidt MF, Wieler LH, Tedin K. Influence of a probiotic *Enterococcus faecium* strain on development of the immune system of sows and piglets. Vet Immunol Immunopathol. 2005;105:151–161. doi:10.1016/j.vetimm.2004.12.022 15797484

[17] Scharek L, Guth J, Filter M, Schmidt MFG. Impact of the probiotic bacteria *Enterococcus faecium* NCIMB 10415 (SF68) and *Bacillus cereus* var. Toyoi NCIMB 40112 on the development of serum IGg and faecal IgA of sows and their piglets. Arch Anim Nutr. 2007;61:223–234. doi:10.1080/17450390701431540 17760301

[18] Canani RB, Cirillo P, Terrin G, Cesarano L, Spagnuolo MI, Vincenzo AD, Albano F, Passariello A, Marco GD, Manguso F, et al. Probiotics for treatment of acute diarrhoea in children: randomised clinical trial of five different preparations. BMJ. 2007;335:340. doi:10.1136/bmj.39272.581736.55 17690340 PMC1949444

[19] Szabó I, Wieler LH, Tedin K, Scharek-Tedin L, Taras D, Hensel A, Appel B, Nöckler K. Influence of a probiotic strain of *Enterococcus faecium* on *Salmonella enterica* serovar Typhimurium DT104 infection in a porcine animal infection model. Appl Environ Microbiol. 2009;75:2621–2628. doi:10.1128/AEM.01515-08 19270131 PMC2681714

[20] Kreuzer S, Janczyk P, Aßmus J, Schmidt MFG, Brockmann GA, Nöckler K. No beneficial effects evident for *Enterococcus faecium* NCIMB 10415 in weaned pigs infected with *Salmonella enterica* serovar Typhimurium DT104. Appl Environ Microbiol. 2012;78:4816–4825. doi:10.1128/AEM.00395-12 22544257 PMC3416376

[21] Siepert B, Reinhardt N, Kreuzer S, Bondzio A, Twardziok S, Brockmann G, Nöckler K, Szabó I, Janczyk P, Pieper R, et al. *Enterococcus faecium* NCIMB 10415 supplementation affects intestinal immune-associated gene expression in post-weaning piglets. Vet Immunol Immunopathol. 2014;157:65–77. doi:10.1016/j.vetimm.2013.10.013 24246154

[22] Elewaut D, DiDonato JA, Kim JM, Truong F, Eckmann L, Kagnoff MF. NF-kappaB is a central regulator of the intestinal epithelial cell innate immune response induced by infection with enteroinvasive bacteria. J Immunol. 1999;163:1457–1466. 10415047

[23] Hayden MS, Ghosh S. NF-κB, the first quarter-century: remarkable progesss and outstanding questions. Genes Dev. 2012;26:203–234. doi:10.1101/gad.183434.111 22302935 PMC3278889

[24] Sun S-C. The non-canonical NF-κB pathway in immunity and inflammation. Nat Rev Immunol. 2017;17:545–558. doi:10.1038/nri.2017.52 28580957 PMC5753586

[25] Barnes AMT, Dale JL, Chen Y, Manias DA, Quaintance KEG, Karau MK, Kashyap PC, Patel R, Wells CL, Dunny GM. *Enterococcus faecalis* readily colonizes the entire gastrointestinal tract and forms biofilms in a germ-free mouse model. Virulence. 2017;8:282–296. doi:10.1080/21505594.2016.1208890 27562711 PMC5411234

[26] Gerdes J. Ki-67 and other proliferation markers useful for immunohistological diagnostic and prognostic evaluations in human malignancies. Semin Cancer Biol. 1990;1:199–206. 2103495

[27] Scholzen T, Gerdes J. The Ki-67 protein: from the known and the unknown. J Cell Physiol. 2000;182:311–322. doi:10.1002/(SICI)1097-4652(200003)182:3<311::AID-JCP1>3.0.CO;2-9 10653597

[28] Christian F, Smith EL, Carmody RJ. The regulation of NF-kappaB subunits by phosphorylation. Cells. 2016;5:12. doi:10.3390/cells5010012 PMC4810097 26999213

[29] Yang Y, Wu J, Wang J. A database and functional annotation of NF-κB target genes. Int J Clin Exp Med. 2016;9:7986–7995.

[30] Zhang Q, Lenardo MJ, Baltimore D. 30 years of NF-κB: a blossoming of relevance to human pathobiology. Cell. 2017;168(1–2):37–57. doi:10.1016/j.cell.2016.12.012 28086098 PMC5268070

[31] Gröbner S, Fritz E, Schoch F, Schaller M, Berger AC, Bitzer M, Autenrieth IB. Lysozyme activates *Enterococcus faecium* to induce necrotic cell death in macrophages. Cell Mol Life Sci. 2010;67(19):3331–3344. doi:10.1007/s00018-010-0384-9 20458518 PMC11115887

[32] Höring S, Schütz M, Autenrieth IB, Gröbner S. Lysozyme facilitates adherence of *Enterococcus faecium* to host cells and induction of necrotic cell death. Microbes Infect. 2012;14(6):554–562. doi:10.1016/j.micinf.2012.01.005 22306275

[33] Li Y, Tong Z, Ling J. Effect of the three *Enterococcus faecalis* strains on apoptosis in MC 3T3 cells. Oral Dis. 2019;25(1):309–318. doi:10.1111/odi.12883 29729070

[34] Chi D, Lin X, Meng Q, Tan J, Gong Q, Tong Z. Real-time induction of macrophage apoptosis, pyroptosis, and necroptosis by *Enterococcus faecalis* OG1RF and two root canal isolated strains. Frontiers in Cellular and Infection Microbiology. 2021;11:720147. doi:10.3389/fcimb.2021.720147 34513732 PMC8427696

[35] Jansen WTM, Bolm M, Balling R, Chhatwal GS, Schnabel R. Hydrogen peroxide-mediated killing of caenorhabditis elegans by *Streptococcus pyogenes*. Infect Immun. 2002;70(9):5202–5207. doi:10.1128/IAI.70.9.5202-5207.2002 12183571 PMC128270

[36] Bolm M, Jansen WTM, Schnabel R, Chhatwal GS. Hydrogen peroxide-mediated killing of *caenorhabditis elegans*: a common feature of different streptococcal species. Infect Immun. 2004;72(2):1192–1194. doi:10.1128/IAI.72.2.1192-1194.2004 14742574 PMC321644

[37] Moy TI, Mylonakis E, Calderwood SB, and Ausubel FM. Cytotoxicity of hydrogen peroxide produced by *Enterococcus faecium*. Infect Immun. 2004;72(8):4512–4520. doi:10.1128/IAI.72.8.4512-4520.2004 15271910 PMC470665

[38] Degnan BA, Palmer JM, Robson T, Jones CED, Fischer M, Glanville M, Mellor GD, Diamond AG, Kehoe MA, Goodacre JA. Inhibition of human peripheral blood mononuclear cell proliferation By *Streptococcus pyogenes* cell extract is associated with arginine deiminase activity. Infect Immun. 1998;66(7):3050–3058. doi:10.1128/IAI.66.7.3050-3058.1998 9632565 PMC108312

[39] Degnan BA, Fontaine MC, Doebereiner AH, Lee JJ, Mastroeni P, Dougan G, Goodacre JA, Kehoe MA. Characterization of an isogenic mutant of *Streptococcus pyogenes* manfredo lacking the ability to make streptococcal acid glycoprotein. Infect Immun. 2000;68(5):2441–2448. doi:10.1128/IAI.68.5.2441-2448.2000 10768929 PMC97444

[40] Cusumano ZT, Watson ME Jr, Caparon MG, Camilli A. *Streptococcus pyogenes* arginine and citrulline catabolism promotes infection and modulates innate immunity. Infect Immun. 2014;82(1):233–242. doi:10.1128/IAI.00916-13 24144727 PMC3911826

[41] Kaur B, Kaur R. Purification of a dimeric arginine deiminase from *Enterococcus faecium* GR7 and study of its anti-cancerous activity. Protein Expression and Purification. 2016;125:53–60. doi:10.1016/j.pep.2015.09.011 26363115

[42] Miyazaki K, Takaku H, Umeda M, Fujita T, Huang W, Kimura T, Yamashita J, Horio T. Potent growth inhibition of human tumor cells in culture by arginine deiminase purified from a culture medium of a Mycoplasma-infected cell line. Cancer Res. 1990;50:4522–4527. 2164440

[43] Somani RR, Chaskar PK. Arginine deiminase enzyme evolving as a potential antitumor agent. Mini Rev Med Chem. 2018;18(4):363–368. doi:10.2174/1389557516666160817102701 27538511

[44] Palmer KL, Godfrey P, Griggs A, Kos VN, Zucker J, Desjardins C, Cerqueira G, Gevers D, Walker S, and Wortman J, et al. Comparative genomics of enterococci: variation in *Enterococcus faecalis*, clade structure in *E.* *faecium*, and defining characteristics of *E.* *gallinarum* and *E. casseliflavus*. mBio. 2012;3(1):e00318–11. doi:10.1128/mBio.00318-11 22354958 PMC3374389

[45] Lauté-Caly DL, Raftis EJ, Cowie P, Hennessy E, Holt A, Panzica DA, Sparre C, Minter B, Stroobach E, Mulder IE. The flagellin of candidate live biotherapeutic *Enterococcus gallinarum* MRx0518 is a potent immunostimulant. Sci Rep. 2019;9(1):801. doi:10.1038/s41598-018-36926-8 30692549 PMC6349862

[46] Kawai T, Akira S. TLR signaling. Cell Death Differ. 2006;13:816–825. 16410796 10.1038/sj.cdd.4401850

[47] Oeckinghaus A, Hayden MS, Ghosh S. Crosstalk in NF-κB signaling pathways. Nat Immunol. 2011;12:695–708. 21772278 10.1038/ni.2065

[48] Rahman MM, McFadden G. Modulation of NF-κB signalling by microbial pathogens. Nat Rev Microbiol. 2011;9:291–306. 21383764 10.1038/nrmicro2539 PMC3611960

[49] Sun S-C, Ganchi PA, Ballard DW, Greene WC. NF-κB controls expression of inhibitor IκBα: evidence for an inducible autoregulatory pathway. Science. 1993;259:1912–1915. 8096091 10.1126/science.8096091

[50] Shembade N, Harhaj EW. Regulation of NF-κB signaling by the A20 deubiquitinase. Cell Mol Immunol. 2012;9:123–130. 22343828 10.1038/cmi.2011.59 PMC3532050

[51] Carlotti F, Dower SK, Qwarnstrom EE. Dynamic shuttling of nuclear factor κB between the nucleus and cytoplasm as a consequence of inhibitor dissociation. J Biol Chem. 2000;275:41028–41034. 11024020 10.1074/jbc.M006179200

[52] Bohuslav J, Chen L-F, Kwon H, Mu Y, Greene WC. p53 induces NF-κB activation by an IκB kinase-independent mechanism involving phosphorylation of p65 by ribosomal S6 kinase 1. J Biol Chem. 2004;279:26115–26125. 15073170 10.1074/jbc.M313509200

[53] Sasaki CY, Barberi TJ, Ghosh P, Longo DL. Phosphorylation of RelA/p65 on serine 536 defines an IκBα-independent NF-κB pathway. J Biol Chem. 2005;280:34538–34547. 16105840 10.1074/jbc.M504943200

[54] Buss H, Handschick K, Jurrmann N, Pekkonen P, Beuerlein K, Müller H, Wait R, Saklatvala J, Ojala PM, Schmitz ML, et al. Cyclin-dependent kinase 6 phosphorylates NF-κB p65 at serine 536 and contributes to the regulation of inflammatory gene expression. PLoS One. 2012;7:e51847. 23300567 10.1371/journal.pone.0051847 PMC3530474

[55] Seki E, Brenner DA, Karin M. A liver full of JNK: signaling in regulation of cell function and disease pathogenesis and clinical approaches. Gastroenterol. 2012;143:307–320. 10.1053/j.gastro.2012.06.004 PMC3523093 22705006

[56] Takaku H, Takase M, Abe S-I, Hayashi H, Miyazaki K. In vivo anti-tumor activity of arginine deiminase purified from *Mycoplasma arginini*. Int J Cancer. 1992;51:244–249. 1568792 10.1002/ijc.2910510213

[57] Phillips MM, Sheaff MT, Szlosarek PW. Targeting arginine-dependent cancers with arginine-degrading enzymes: opportunities and challenges. Cancer Res Treat. 2013;45:251–262. 24453997 10.4143/crt.2013.45.4.251 PMC3893322

[58] Xiong L, Teng JL, Botelho MG, Lo RC, Lau SK, Woo PC. Arginine metabolism in bacterial pathogenesis and cancer therapy. Int J Mol Sci. 2016;17:363. 26978353 10.3390/ijms17030363 PMC4813224

[59] Ren W, Rajendran R, Zhao Y, Tan B, Wu G, Bazer FW, Zhu G, Peng Y, Huang X, Deng J, et al. Amino acids as mediators of metabolic cross talk between host and pathogen. Front Immunol. 2018;9:319. doi:10.3389/fimmu.2018.00319 29535717 PMC5835074

[60] Jung J, Genau HM, Behrends C. Amino acid-dependent mTORC1 regulation by the lysosomal membrane protein SLC38A9. Mol Cell Biol. 2015;35:2479–2494. 25963655 10.1128/MCB.00125-15 PMC4475919

[61] Chantranupong L, Scaria SM, Saxton RA, Gygi MP, Shen K, Wyant GA, Wang T, Harper JW, Gygi SP, Sabatini DM. The CASTOR proteins are arginine sensors for the mTORC1 pathway. Cell. 2016;165:153–164. 26972053 10.1016/j.cell.2016.02.035 PMC4808398

[62] Wyant GA, Abu-Remaileh M, Wolfson RL, Chen WW, Freinkman E, Danai LV, Van der Heiden MG, Sabatini DM. mTORC1 activator SLC38A9 is required to efflux essential amino acids from lysosomes and use protein as a nutrient. Cell. 2017;171:642–654. 29053970 10.1016/j.cell.2017.09.046 PMC5704964

[63] Bar-Peled L, Sabatini DM. Regulation of mTORC1 by amino acids. Trends Cell Biol. 2014;24:400–406. 24698685 10.1016/j.tcb.2014.03.003 PMC4074565

[64] Saxton RA, Sabatini DM. mTOR signaling in growth, metabolism, and disease. Cell. 2017;168:960–976. 28283069 10.1016/j.cell.2017.02.004 PMC5394987

[65] Yao Y, Jones E, Inoki K. Lysosomal regulation of mTORC1 by amino acids in mammalian cells. Biomolecules. 2017;7:51. doi:10.3390/biom7030051 PMC5618232 28686218

[66] Casiano-Colon A, Marquis RE. Role of arginine deiminase system in protecting oral bacteria and an enzymatic basis for acid tolerance. Appl Environ Microbiol. 1988;54:1318–1324. 2843090 10.1128/aem.54.6.1318-1324.1988 PMC202656

[67] Ryan S, Begley M, Gahan CGM, Hill C. Molecular characterization of the arginine deiminase system in *Listeria monocytogenes*: regulation and role in acid tolerance. Environ Microbiol. 2009;11:432–445. 19196274 10.1111/j.1462-2920.2008.01782.x

[68] Choi Y, Choi J, Groisman EA, Kang D-H, Shin D, Ryu S. Expression of STM4467-encoded arginine deiminase controlled by the STM4463 regulator contributes to *Salmonella enterica* serovar Typhimurium virulence. Infect Immun. 2012;80:4291–4297. 23006851 10.1128/IAI.00880-12 PMC3497419

[69] Tattoli I, Sorbara MT, Vuckovic D, Ling A, Soares F, Carneiro LAM, Yang C, Emili A, Philpott D, Girardin SE. Amino acid starvation induced by invasive bacterial pathogens triggers an innate host defense program. Cell Host Microbe. 2012;11:563–575. 22704617 10.1016/j.chom.2012.04.012

[70] Abdel-Nour M, Tsalikis J, Kleinman D, Girardin SE. The emerging role to mTOR signalling in antibacterial immunity. Immunol Cell Biol. 2014;92:346–353. 24518980 10.1038/icb.2014.3

[71] Nouwen LV, Everts B. Pathogens menTORing Macrophages and dendritic cells: manipulation of mTOR and cellular metabolism to promote immune escape. Cells. 2020;9:161. doi:10.3390/cells9010161 PMC7017145 31936570

[72] Ye Jin L, Choi HJ, Kang TW, Kim HO, Chung MJ, Park YM. CBT-SL5, a bacteriocin from *Enterococcus faecalis*, suppresses the expression of interleukin-8 induced by *Propionibacterium acnes* in cultured human keratinocytes. J Microbiol Biotechnol. 2008;18:1308–1316. 18667861

[73] Wang S, Hibberd ML, Pettersson S, Lee YK. *Enterococcus faecalis* from healthy infants modulates inflammation through MAPK signaling pathways. PLoS One. 2014;9:e97523. 24830946 10.1371/journal.pone.0097523 PMC4022717

[74] Zou J, Shankar N. Surface protein Esp enhances pro-inflammatory cytokine expression through NF-κB activation during enterococcal infection. Innate Immun. 2016;22:31–39. 26503704 10.1177/1753425915611237

[75] Tian Z, Yang L, Li P, Xiao Y, Peng J, Wang X, Li Z, Liu M, Bi D, Shi D. The inflammation regulation effects of *Enterococcus faecium* HDRsEf1 on human enterocyte-like HT-29 cells. Anim Cells Syst. 2016;20:70–76.

[76] Cuív PO, Giri R, Hoedt EC, McGuckin MA, Begun J, Morrison M. *Enterococcus faecalis* AHG0090 is a genetically tractable bacterium and produces a secreted peptidic bioactive that suppresses nuclear factor kappa B activation in human gut epithelial cells. Front Immunol. 2018;9:790. doi:10.3389/fimmu.2018.00790 29720977 PMC5915459

[77] Winterhoff N, Goethe R, Gruening P, Rohde M, Kalisz H, Smith HE, Valentin-Weigand P. Identification and characterization of two temperature-induced surface-associated proteins of *Streptococcus suis* with high homologies to members of the arginine deiminase system of *Streptococcus pyogenes*. J Bacteriol. 2002;184:6768–6776. 12446626 10.1128/JB.184.24.6768-6776.2002 PMC135470

[78] Zoncu R, Bar-Peled L, Efeyan A, Wang S, Sancak Y, Sabatini DM. mTORC1 senses lysosomal amino acids through an inside-out mechanism that requires the vacuolar H^+^-ATPase. Science. 2011;334:678–683. 22053050 10.1126/science.1207056 PMC3211112

[79] Rebsamen M, Pochini L, Stasyk T, de Araújo MEG, Galluccio M, Kandasamy RK, Snijder B, Fauster A, Rudashevskaya EL, Bruckner M, et al. SLC38A9 is a componenet of the lysosomal amino acid sensing machinery that controls mTORC1. Nature. 2015;519:477–481. 25561175 10.1038/nature14107 PMC4376665

[80] Dan HC, Cooper MJ, Cogswell PC, Duncan JA, Ting JP-Y, Baldwin AS. Akt-dependent regulation of NF-κB is controlled by mTOR and raptor in association with IKK. Genes Dev. 2008;22:1490–1500. 18519641 10.1101/gad.1662308 PMC2418585

[81] Wong C-O, Palmieri M, Li J, Akhmedov D, Chao Y, Broadhead GT, Zhu MX, Berdeaux R, Collins CA, Sardiello M, et al. Diminished mTORC1-dependent JNK activation underlies the neurodevelopmental defects associated with lysosomal dysfunction. Cell Rep. 2015;12:2009–2020. 26387958 10.1016/j.celrep.2015.08.047 PMC4591237

[82] Gajanayaka N, Dong SXM, Ali H, Iqbal S, Mookerjee A, Lawton DA, Caballero RE, Cassol E, Cameron DW, Angel JB, et al. TLR-4 agonist induces IFN-γ production selectively in proinflammatory human M1 macrophages through the PI3K-mTOR- and JNK-MAPK-activated p70S6K pathway. J Immunol. 2021;207:2310–2324. 34551966 10.4049/jimmunol.2001191

