# Supplementary material for: A virulence factor as a therapeutic: the probiotic Enterococcus faecium SF68 arginine deiminase inhibits innate immune signaling pathways
Source: Gut Microbes. 2022 Aug 3;14(1):2106105. doi: 10.1080/19490976.2022.2106105 (PMC9351580; doi:10.1080/19490976.2022.2106105)
Supplement: Supplemental Material [file KGMI_A_2106105_SM4272.zip › Manuscript Ghazisaeedi et al Suppl Figures.pdf]

Supplementary Figures Ghazisaeedi *et al*., 2022

Fig. S1

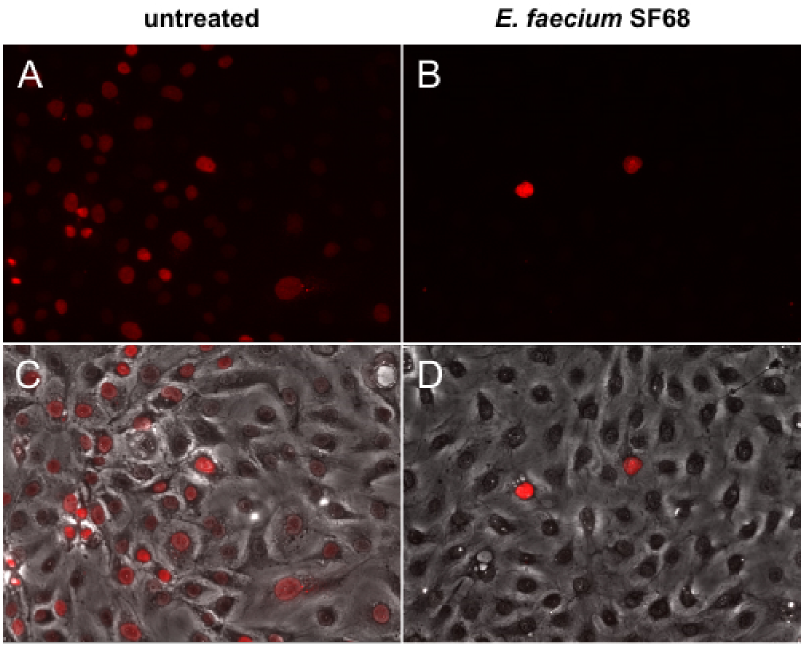

Supplementary Fig. S1. IPEC-J2 cells treated with *E. faecium* SF68 cease to proliferate, but do not show signs of cytotoxicity. Cell cultures of the IPEC-J2 cell line were grown to near confluency, followed by an exchange of cell culture medium with either fresh medium (A, C), or medium containing bacterial lysates of *E. faecium* SF68 (B, D), and incubated for an additional 24 h. The following day, cells were stained for the presence of the proliferation marker Ki67. Shown are fluorescence micrographs for Ki67 staining (A, B) and an overlay of immunofluorescence and phase-contrast micrographs for the same microscopic fields (C, D). The results shown are representative of two, independent experiments with three, replicate cover slips each.

Fig. S2

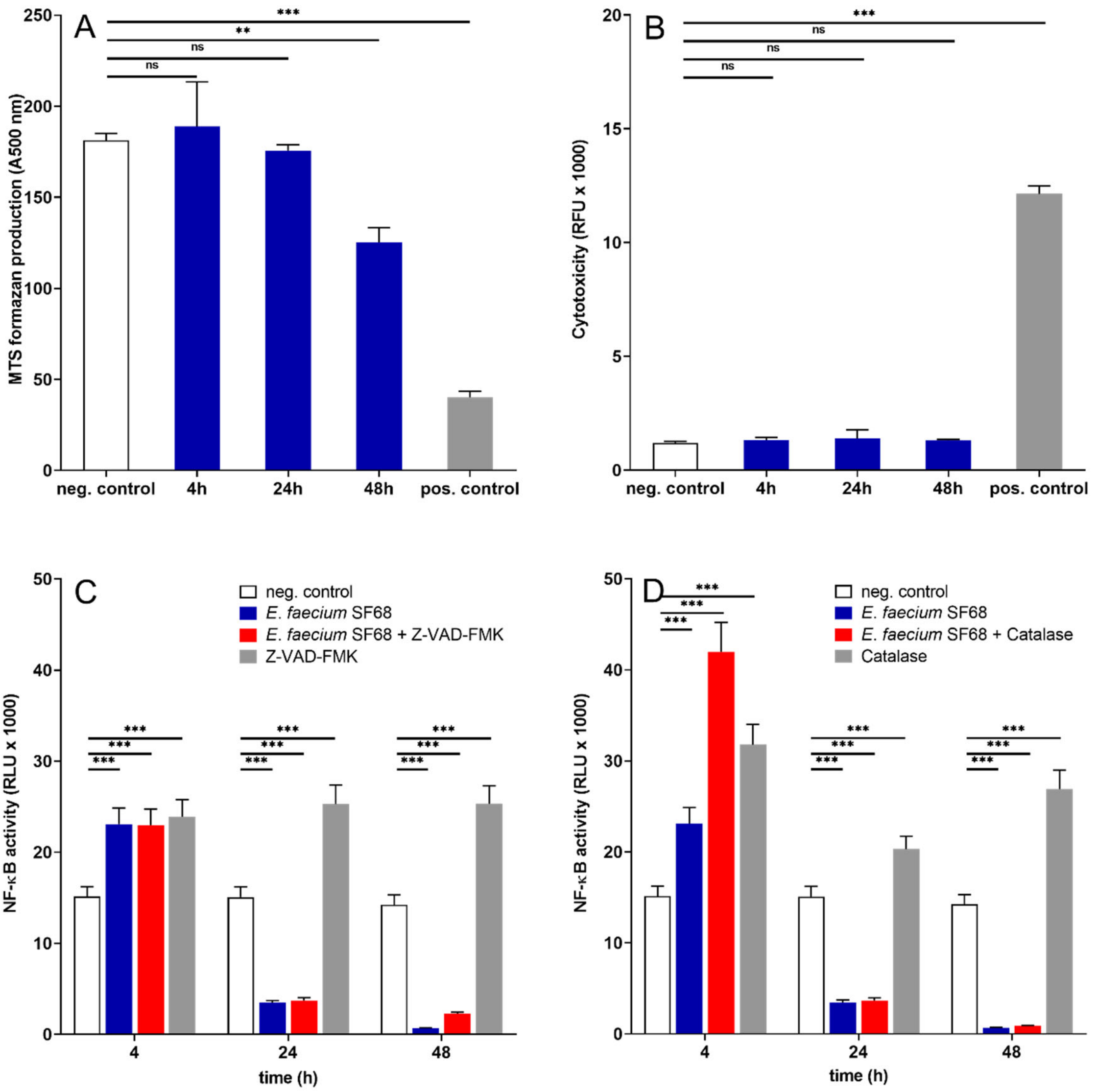

Supplementary Fig. S2. Cells treated with *E. faecium* SF68 do not show signs of loss of viability, cytotoxicity, apoptosis, or sensitivity to hydrogen peroxide. (A) Confluent monolayers were treated with *E. faecium* SF68 lysates and at the times indicated, samples were taken for determination of cell viability. Untreated control cells are indicated by the open bar, the positive control (ionomycin treatment) by the grey bar. (B) Confluent monolayers were treated as in (A), and at the times indicated, cells were examined for cytotoxicity as determined by LDH release. Untreated wells (open

bar) or wells treated with saponin (grey bar) served as negative and positive controls, respectively. (C) Cells were either left untreated (open bars), treated with *E. faecium* SF68 lysates (blue bars), the caspase inhibitor Z-VAD-FMK (grey bars), or *E. faecium* SF68 lysates in the presence of Z-VAD-FMK (red bars), and replicate wells sampled for NF-κB activity (luciferase) at the times indicated below the graph. (D) Untreated cells (open bars), cells treated with *E. faecium* SF68 lysates (blue bars), 2500 U catalase (grey bars), or *E. faecium* SF68 lysates in the presence of catalase (red bars), were incubated for the times indicated below the graph, and replicate wells were sampled for NF-κB activity. For all assays, the results shown are representative of at least two, independent experiments.

Fig. S3

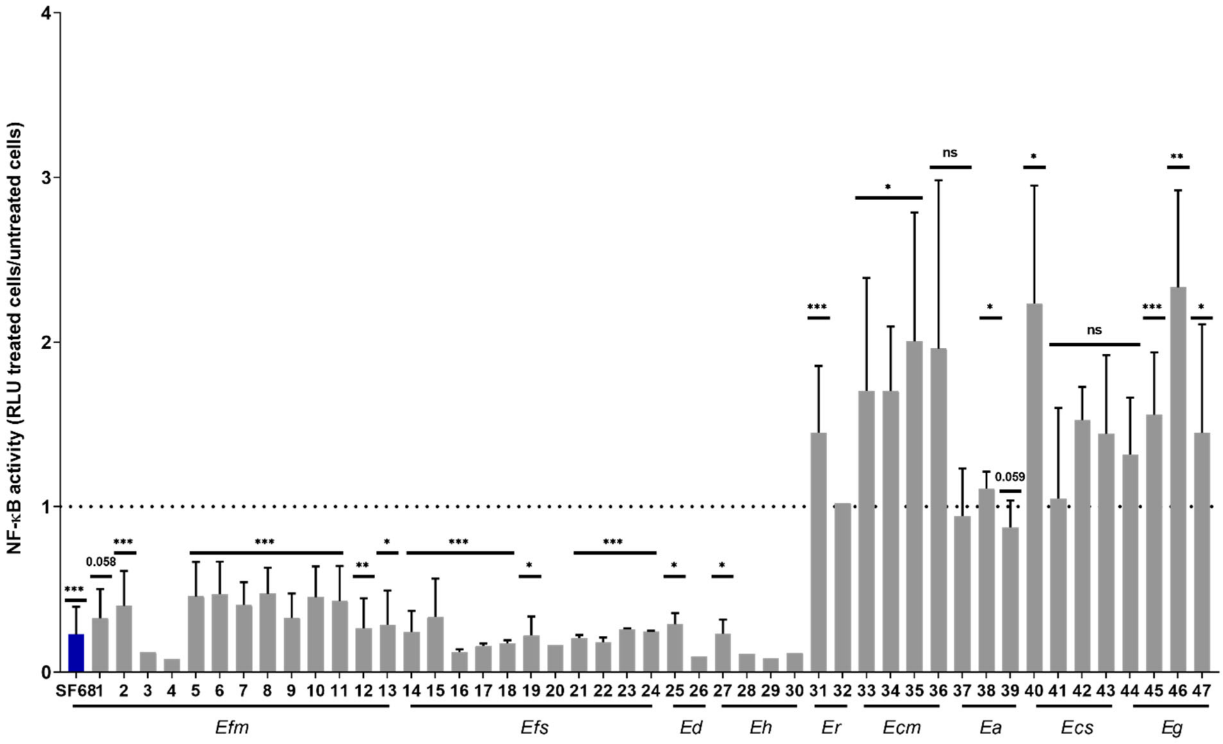

Supplementary Fig. S3. Inhibition of NF-κB activity is *Enterococcus* species-specific. Confluent monolayers of the IPEC-J2/K6 NF-κB luciferase reporter cell line were incubated in the presence of 5 µg of total protein of bacterial lysates of either *E. faecium* SF68 (blue bar) or other representative *Enterococcus* species (grey bars) as indicated below the graph for 24 h followed by determination of the relative NF-κB (luciferase) activity compared to untreated, control cells determined in parallel (dotted line). SF68, *E. faecium* SF68; Efm, *E. faecium* isolates; Efs, *E. faecalis* isolates; Ed, *E. durans*; Eh, *E. hirae*; Er, *E. raffinosus*; Ecm, *E. cecorum*; Ea, *E. avium*; Ecs, *E. casseliflavus*; Eg, *E. gallinarum*. See supplementary Table S2 for additional strain information for each isolate. Where the statistical significance is shown, the results are the averages of at least three, independent assays for each *Enterococcus* isolate. Where no significance is shown, the experiments were performed only once and are only confirmatory.

Fig. S4

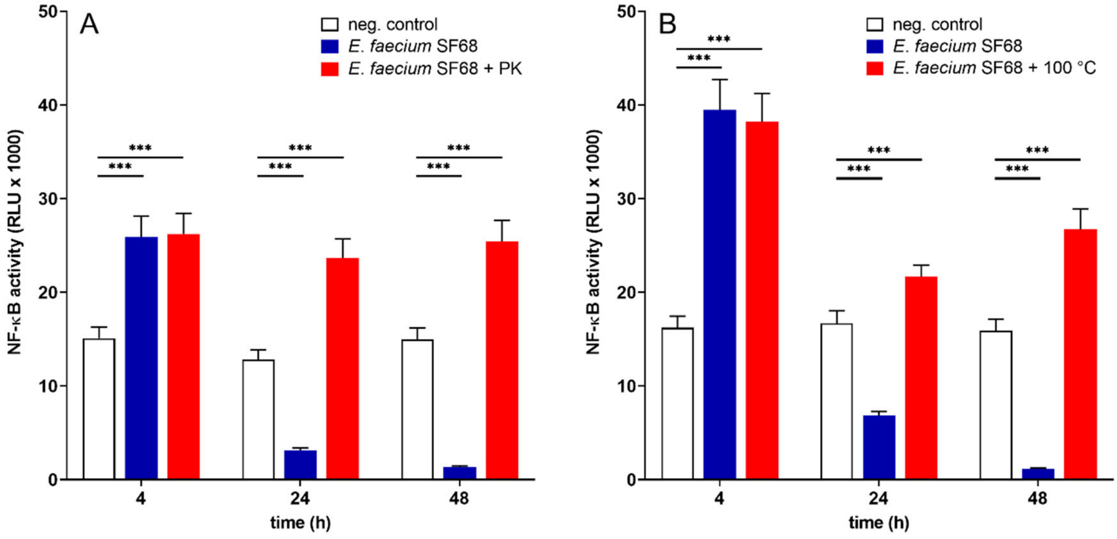

Supplementary Fig. S4. The NF-κB inhibitory factor of *E. faecium* SF68 is proteinaceous in nature. The porcine, IPEC-J2/K6 intestinal epithelial cell line harboring an NF-κB responsive luciferase reporter was incubated for the times indicated in the presence of cell-free, whole cell bacterial lysates of *E. faecium* SF68 (blue bars), or with lysates pre-treated with either (A) proteinase K (red bars), or (B) heated to 100°C prior to addition to the cell cultures (red bars). At the times indicated below the graphs, the NF-κB-dependent luciferase activity in replicate wells was determined. In all panels, the basal levels of NF-κB activity of untreated cells at each time point are indicated by open bars. The results shown are the averages of two, independent assays.
