# Supplementary material for: A virulence factor as a therapeutic: the probiotic Enterococcus faecium SF68 arginine deiminase inhibits innate immune signaling pathways
Source: Gut Microbes. 2022 Aug 3;14(1):2106105. doi: 10.1080/19490976.2022.2106105 (PMC9351580; doi:10.1080/19490976.2022.2106105)
Supplement: Supplemental Material [file KGMI_A_2106105_SM4272.zip › Manuscript Ghazisaeedi et al Suppl Methods.pdf]

Electroporation of E. avium. Electroporation of *E. avium* with plamids with pMGS100 (1) and pMGS100-$arcA_{SF68}^{+}$ was performed as previously described (2) with modifications. Briefly, overnight cultures of *E. avium* strain IMT39925 grown in LB medium containing 5% (v/v) glycine and 0.5 M sucrose were inoculated at a dilution of 1:100 into the same, fresh medium and incubated with aeration at 37°C for 5-6 h. Bacteria were collected by centrifugation for 15 min. at 4°C and 10000 x *g*. The supernatants were discarded, and the bacterial pellets were resuspended in 5 ml of ice-cold, 10% (v/v) glycerol, and brought to 10 ml with an additional 5 ml of ice-cold, 10% glycerol. The bacterial pellets were collected by centrifugation again for 10 min., resuspended in ice-cold, 10% glycerol, and collected a third time by centrifugation for 10 min. at 4°C and 6000 x *g*. The washing steps were repeated again, and the bacterial pellets collected by centrifugation for 10 min. at 4°C and 3000 x *g*. Finally, the resuspended bacteria were collected by centrifugation for 10 min. at 4°C and 1500 x *g*, the supernatant was discarded, and the bacterial pellets were resuspended in 0.2 ml of ice-cold, 10% glycerol, and 0.1 ml aliquots were frozen at -70°C overnight. The bacterial suspensions and pellets were maintained throughout on ice.

The electroporation of *E. avium* electrocompetent cells with plasmids pMGS100 or pMGS100-$arcA_{SF68}^{+}$ was performed by addition of electrocompetent cells to pre-cooled, 2 mm gap, electroporation cuvettes (VWR) on ice, to which plasmid DNA was added at various concentrations. Cells were electroporated in a GenePulser XCell instrument (BioRad) at a setting of 25 µF, 2.5 kV, and 200 Ω. Immediately following the electroporation, 0.5 ml of fresh LB medium was added directly into the cuvette, and the bacterial suspensions were transferred to 1.5 ml reaction tubes and incubated at 37°C for 30-45 min., followed by plating of 10 µl to 100 µl aliquots to LB agar plates containing 20 µg/ml chloramphenicol, and incubated at 37°C overnight. Chloramphenicol-resistant colonies were inoculated into LB medium containing chloramphenicol, and bacterial lysates of putative clones

were subjected to PCR screening with primers specific for either the vector or the cloned *E. faecium* SF68 *arcA* gene. Positive clones were further subjected to sequencing for verification.

Ammonium sulfate fractionation of Enterococcus proteins

Ammonium sulfate precipitation/fractionation of proteins in bacterial lysates was performed using standard protocols, essentially as previously described (3). Briefly, overnight cultures of *E. faecium* SF68 and *E. avium* strains grown in brain heart infusion (BHI) broth were used to inoculate two, 300 ml volumes of BHI broth and grown at 37°C with aeration. At an optical density at 600 nm (OD_{600}) of approximately 1.8 (≈ 2 x 10^9 CFU/ml), the bacteria were collected by centrifugation for 15 min, at 4°C and 10000 x *g*, in a GSA rotor. The resulting bacterial pellets were each resuspended in 50 ml of 1X phosphate buffered saline (PBS) and centrifuged again for 10 min. at 4°C, 10000 x *g*, and the resulting bacterial pellets were frozen at -20°C. After resuspension of the bacterial pellets in a total of 10 ml of ice-cold 1X PBS, the concentrated bacterial suspensions were lysed with five passages through a French press at 18000 psi. The resulting bacterial cell lysates were cleared by centrifugation for 30 min at 4°C, 10000 x *g*. The supernatants were collected, and 10 ml was subjected to ammonium sulfate (AS) fractionation in steps of 30%, 60% and 100% (w/v) ammonium sulfate solutions by addition of ammonium sulfate to 30% saturation with stirring on ice for approximately 30 min., followed by an additional 90 min. on ice. Precipitated proteins of the 30% saturated AS fraction were collected by centrifugation for 30 min., at 4°C, at 10000 x *g*. The resulting supernatnants were collected for the subsequent 60% and 100% fractionations (as above) by addition of the appropriate amounts of ammonium sulfate to 10 ml of supernatant of the previous fractions. The pellets of the AS fractions were resuspended in 0.5 ml (30% AS fraction) or 1.0 ml (60% and 100% fractions) of 1X PBS. Protein concentrations of the initial French press lysate and subsequent supernatant and pellet resuspensions were performed using BCA assays.

Determination of arginine deiminase (ADI) activity

Arginine deiminase (ADI) activities in the AS fractions of *E. faecium* SF68 and *E. avium* were determined from 25 µl, 50 µl, and 100 µl aliquots of 1:5 dilutions in water of the French press and AS fractions and supernatants brought to a final volume of 100 µl in water and added to 400 µl of 0.1 M potassium phosphate buffer, pH 6.5, containing 10 mM L-arginine, for a final total volume of 500 µl. The reactions were incubated at 37°C for 2 h, and terminated by addition of 250 µl of a stop solution consisting of 96% sulfuric acid and 85% orthophosphoric acid. A volume of 31.3 µl of 3% diacetyl monoxim was added to each reaction mix, and the samples were boiled for 15 minutes at 100°C in the dark. The reactions were allowed to cool to room temperature for 10 min. in the dark, and the ADI activity of the samples was determined by the absorption at 440 nm. Reactions without addition of bacterial lysate or AS fractions served as negative (background) controls. The ADI activity of the samples was determined by extrapolation from the linear range of values of a standard curve of absorption at 440 nm of 0 to 100 µg of citrulline prepared in parallel. The final ADI activity was calculated as the amount of citrulline in µg generated/µg added protein/h and converted to nmol citrulline/h/mg protein as previously described (4).

MALDI-TOF identification of ammonium sulfate fraction proteins

Proteins present in the different AS fractions of *E. faecium* and *E. avium* lysates were separated using sodium dodecyl sulphate polyacrylamide gel electrophoresis (SDS-PAGE) followed by visualization using silver staining. The SDS-PAGE bands present in the active ammonium sulfate fraction of *E. faecium* SF68 lysate, which were not present in other fractions of the *E. faecium* SF68 lysate or the same AS fraction of *E. avium* lysates, were excised from the gel, destained and digested with sequencing grade trypsin at 100 µg/ml (Promega) as previously described (5). Digested peptides were spotted onto a ground steel MTP 384 MALDI target plate (Bruker Daltonics, Germany), using the dried-droplet technique and α-Cyano-4-hydroxycinnamicacid (HCCA) (Sigma-Aldrich, Germany) matrix. Protein identification was carried out using matrix-assisted laser desorption ionization with

an Ultraflex II TOF/TOF time-of-flight mass spectrometer (MALDI-TOF MS; Bruker Daltonics) as described (6).

Quantitative real-time PCR of IL-6, IL-8, and Bax gene expression

Real-time PCR (RT-PCR) for cytokine/chemokine (IL-6, IL-8), and apoptosis (Bax) gene expression were performed using SYBR Green SensiFAST Probe Lo-ROX Master Mix (Bioline, UK) and a StepOnePlus™ Real-time PCR System (Applied Biosystems). 20 μL of final volume including 2 μL of cDNA (1:10 dilution) template was added for each sample to a MicroAmp™ 96-Well Reaction Plate (Thermo Fisher) and amplified in duplicate using gene-specific primer pairs for porcine β-actin, IL-6, IL-8, and Bax genes. Sequences for the primers were based on prior publications (7-10) and are listed in supplementary Table S3. Results were normalized to the housekeeping gene β-actin. β-Actin was chosen as the reference gene based on prior publications showing high stability in a range of porcine tissues based on geNorm, BestKeeper, and NormFinder software analyses of qRT-PCR (11,12), as well as our own determinations comparing β-Actin and GAPDH (13). The relative changes in gene expression was determined using the $2^{-\Delta\Delta Ct}$ method (14), with relative gene expression indicated as -fold change relative to untreated, control cells.

References

1. Fujimoto S, Ike Y. pAM401-based shuttle vectors that enable overexpression of promoterless genes and one-step purification of tag fusion proteins directly from *Enterococcus faecalis*. *Appl Environ Microbiol* 2001; 67:1262-1267.
2. Cruz-Rodz AL, Gilmore MS. High efficiency introduction of plasmid DNA into glycine treated *Enterococcus faecalis* by electroporation. *Mol Gen Genet*. 1990; 224:152-154.
3. Wingfield PT. Protein precipitation using ammonium sulfate. *Curr Protoc Protein Sci*. 2016; 84:A.3F.1-A.3F.9.
4. Oginsky EL. Isolation and determination of arginine and citrulline. *Methods Enzymol*. 1957; 3:639-643.
5. Shevchenko A, Wilm M, Vorm O, Mann M. Mass spectrometric sequencing of proteins from silver-stained polyacrylamide gels. *Anal Chem*. 1996; 68:850-858.
6. Murugaiyan J, Weise C, von Bergen M, Roesler U. Two-dimensional proteome reference map of *Prototheca zopfii* revealed reduced metabolism and enhanced signal transduction as adaptation to an infectious life style. *Proteomics*. 2013; 13:2664-2669.
7. Dozois CM, Oswald E, Gautier N, Serthelon JP, Fairbrother JM, Oswald IP. A reverse transcription-polymerase chain reaction method to analyze porcine cytokine gene expression. *Vet Immunol Immunopathol*. 1997; 58:287-300.

8. Schierack P, Nordhoff M, Pollmann M, Weyrauch KD, Amasheh S, Lodemann U, Jores J, Tachu B, Kleta S, Blikslager A, et al. Characterization of a porcine intestinal epithelial cell line for *in vitro* studies of microbial pathogenesis in swine. *Histochem Cell Biol*. 2006;125:293-305.
9. Collado-Romero M, Arce C, Ramírez-Boo M, Carvajal A, Garrido JJ. Quantitative analysis of the immune response upon *Salmonella* Typhimurium infection along the porcine intestinal gut. *Vet Res*. 2010; 41:23. doi: 10.1051/vetres/2009072.
10. Yuan B, Liang S, Jin Y-X, Kwon J-W, Zhang J-B, Kim N-H. Progesterone influences cytoplasmic maturation in porcine oocytes developing *in vitro*. *Peer J*. 2016; 4:e2454.
11. Nygard A-B, Jorgensen CB, Cirera S, Fredholm M. Selection of reference genes for gene expression studies in pig tissues using SYBR green qPCR. *BMC Mol Biol*. 2007; 8:67-72.
12. McCulloch RS, Ashwell MS, O´Nan AT, Mente PL. Identification of stable normalization genes for quantitative real-time PCR in porcine articular cartilage. *J Anim Sci Biotech*. 2012; 3:36-42.
13. Siepert B, Reinhardt N, Kreuzer S, Bondzio A, Twardziok S, Brockmann G, Nöckler K, Szabó I, Janczyk P, Pieper R, *et al*. *Enterococcus faecium* NCIMB 10415 supplementation affects intestinal immune-associated gene expression in post-weaning piglets. *Vet Immunol Immunopathol*. 2014; 157:65-77.
14. Livak KJ, Schmittgen TD. Analysis of relative gene expression data using real-time quantitative PCR and the $2^{-\Delta\Delta Ct}$ method. *Methods*. 2001; 25:402-408.
