# Supplementary material for: A virulence factor as a therapeutic: the probiotic Enterococcus faecium SF68 arginine deiminase inhibits innate immune signaling pathways
Source: Gut Microbes. 2022 Aug 3;14(1):2106105. doi: 10.1080/19490976.2022.2106105 (PMC9351580; doi:10.1080/19490976.2022.2106105)
Supplement: Supplemental Material [file KGMI_A_2106105_SM4272.zip › Manuscript Ghazisaeedi et al Suppl TableS1.pdf]

Ghazisaeedi *et al*., 2022. Supplementary Table S1

Bacterial strains, plasmids, and cell lines used in this study

Strain ID	Strain/Isolate*	Source/Reference
IMT39924	*Enterococcus avium* (UW6277)	G Werner, RKI
IMT39925	*Enterococcus avium* (UW11197)	G Werner, RKI
IMT39926	*Enterococcus avium* (UW14640)	G Werner, RKI
IMT16264	*Enterococcus cecorum* (1492/08)	This study
IMT19044	*Enterococcus cecorum* (1644/09)	This study
IMT19051	*Enterococcus cecorum* (1645/09)	This study
IMT21482	*Enterococcus cecorum* (1058)	This study
IMT12358	*Enterococcus casseliflavus* (1860/06)	This study
IMT39927	*Enterococcus casseliflavus* (UW13404)	G Werner, RKI
IMT39928	*Enterococcus casseliflavus* (UW13463)	G Werner, RKI
IMT39929	*Enterococcus casseliflavus* (UW13577)	G Werner, RKI
IMT10686	*Enterococcus durans* (K435/05-1)	This study
IMT38978	*Enterococcus durans*	This study
IMT1318	*Enterococcus faecalis* DSM 2570 (ATCC 29212)	DSMZ
IMT4820	*Enterococcus faecalis* ATCC 14506	ATCC
IMT8076	*Enterococcus faecalis* (K37/03)	This study
IMT8081	*Enterococcus faecalis* (K417/03-5)	This study
IMT8966	*Enterococcus faecalis* (2508/03)	This study
IMT9145	*Enterococcus faecalis* (188-1/04)	This study
IMT9900	*Enterococcus faecalis* (K1055/04-4)	This study
IMT10680	*Enterococcus faecalis* (K1082/04)	This study
IMT11076	*Enterococcus faecalis* (1607/05)	This study
IMT14200	*Enterococcus faecalis* (2021/07)	This study
IMT21655	*Enterococcus faecalis* (1211-1/10)	This study
IMT2059	*Enterococcus faecium* ATCC 6057	DSMZ
4258	*Enterococcus faecium* SF68 (NCIMB 10415, Cylactin®, Cernelle 68®)	Cerbios-Pharma
4259	*Enterococcus faecium* TX16 (TX0016, TEX16, TX16, DO)	BE Murray (1,2)
IMT4823	*Enterococcus faecium* DSM 2918	DSMZ
IMT4824	*Enterococcus faecium*	This study

8094	*Enterococcus faecium* E980	M de Been (3)
8095	*Enterococcus faecium* E1604 (EnGen0028)	M de Been (4)
8096	*Enterococcus faecium* E1861	M de Been (4)
8097	*Enterococcus faecium* E3548	M de Been (4)
8098	*Enterococcus faecium* TX1310	BE Murray
8099	*Enterococcus faecium* TX1330	BE Murray (2)
8100	*Enterococcus faecium* TX2050	BE Murray
IMT8703	*Enterococcus faecium* (K996/03-1)	This study
IMT30343	*Enterococcus faecium* (1593-5/12)	This study
IMT12257	*Enterococcus gallinarum* (L176/06-1)	This study
IMT12258	*Enterococcus gallinarum* (L176/06-2)	This study
IMT12261	*Enterococcus gallinarum* (L179/06-1)	This study
IMT12262	*Enterococcus gallinarum* (L179/06-2)	This study
IMT4819	*Enterococcus hirae* ATCC 9790	ATCC
IMT4821	*Enterococcus hirae* ATCC 8043	ATCC
IMT38123	*Enterococcus hirae* ATCC 10541	ATCC
IMT48802	*Enterococcus hirae* (296-2/20)	This study
IMT23827	*Enterococcus raffinosus* (2019-3/10)	This study
IMT48810	*Enterococcus raffinosus* (276-6/20)	This study
145	*Escherichia coli* K-12 MG1655	CA Gross
1714	*Escherichia coli* K-12 DH5α	Laboratory stock
IMT28316	*Staphylococcus aureus* ATCC 29213	ATCC
Plasmids	Relevant features	Source/Reference
pMGS100	*E. coli*/*E. faecalis* shuttle vector; P*bacA cat*	S Fujimoto (5)
pMGS100-ArcA	P*bacA*-$arcA_{SF68}^{+}$ *cat*	This study
Cell Lines	Relevant features	Source/Reference
Caco-2	human, intestinal epithelia, ATCC HTB-37	ATCC (6)
Caco-2/C6	Caco-2 NF-κB-luciferase reporter Pur^R	This study
IPEC-J2	porcine, intestinal epithelia	A Blikslager (7)
IPEC-J2/K6	IPEC-J2 NF-κB-luciferase reporter Pur^R	This study
IPEC-J2/D6	IPEC-J2 JNK(AP-1)-luciferase reporter Pur^R	This study
MODE-K	murine, intestinal epithelia	D Kaiserlian (8)
MODE-K/H8	MODE-K NF-κB-luciferase reporter Pur^R	This study

*Abbreviations: ATCC, American Type Culture Collection; DSMZ, Deutsche Sammlung von Mikroorganismen und Zellkulturen, Braunschweig, Germany; NCIMB, National Collection of Industrial, Food and Marine Bacteria, Aberdeen Scotland, UK; RKI, Robert Koch Institute. Abbreviations: *cat*, chloramphenicol resistance; Pur^R, puromycin resistance. Additional internal source or laboratory designations for isolates are indicated in parentheses.

References

1. Arduino, RC, Murray BE, Rakita RM. Roles of antibodies and complement phagocytic killing of enterococci. *Infect Immun*. 1994; 62:987-993. (TX0016, TEX16)
2. Qin, X, Galloway-Peña JR, Sillanpaa J, Roh JH, Nallapareddy SR, Chowdhury S, Bourgogne A, Choudhury T, Muzny DM, Buhay CJ, *et al*. Complete genome sequence of *Enterococcus faecium* strain TX16 and comparative genomic analysis of *Enterococcus faecium* genomes. *BMC Microbiology*. 2012; 12:135. http://www.biomedcentral.com/1471-2180/12/135.
3. van Shaik W, Top J, Riley DR, Boekhorst J, Vrijenhoek JE, Schapendonk CM, Hendrickx AP, Nijman IJ, Bonten MJ, Tettelin H, *et al*. Pyrosequencing-based comparative genome analysis of the nosocomial pathogen *Enterococcus faecium* and identification of a large transferable pathogenicity island. *BMC Genomics*. 2010; 11:239. https://doi.org/10.1186/1471-2164-11-239.
4. Lebreton, F, van Schaik W, McGuire AM, Godfrey P, Griggs A, Mazumdar V, Corander J, Cheng L, Saif S, Young S, *et al*. Emergence of epidemic multidrug-resistant *Enterococcus faecium* from animal and commensal strains. *mBio*. 2013; 4:e00534-13.
5. Fujimoto S, Ike Y. pAM401-based shuttle vectors that enable overexpression of promoterless genes and one-step purification of tag fusion proteins directly from *Enterococcus faecalis*. *Appl Environ Microbiol* 2001; 67:1262-1267.
6. Fogh J, Wright, WC, Loveless JD. Absence of HeLa cell contamination in 169 cell lines derived from human tumors. *J. Natl. Cancer Inst*. 1977; 58: 209-214.
7. Berschneider H. Development of normal cultured small intestinal epithelial cell lines which transport Na and Cl. Abstract of the Annual Meeting of the American Gastroenterological Association. 1989; A41.
8. Vidal, K, Grosjean, I, Revillard, J-P, Gespach, C, Kaiserlian, D. Immortalization of mouse intestinal epithelial cells by the SV40-large T gene. *J Immunol Meth* 1993; 166:63-73.
