# Supplementary material for: A virulence factor as a therapeutic: the probiotic Enterococcus faecium SF68 arginine deiminase inhibits innate immune signaling pathways
Source: Gut Microbes. 2022 Aug 3;14(1):2106105. doi: 10.1080/19490976.2022.2106105 (PMC9351580; doi:10.1080/19490976.2022.2106105)
Supplement: Supplemental Material [file KGMI_A_2106105_SM4272.zip › Manuscript Ghazisaeedi et al Suppl TableS2.pdf]

Ghazisaeedi *et al.*, 2022. Supplementary Table S2

Enterococcus strains and isolates used in Supplementary Fig. S1

Nr.	Strain ID	Strain/Isolate*	ADI	Source/Reference
SF68	4258	*Enterococcus faecium* SF68 (NCIMB10415, Cylactin, Cernelle 68)	+	NCIMB
1	IMT2059	*Enterococcus faecium* ATCC 6057	+	DSMZ
2	4259	*Enterococcus faecium* TX16 (TX0016, TEX16, DO)	+	BE Murray (1,2)
3	IMT4823	*Enterococcus faecium* DSM 2918	+	DSMZ
4	IMT4824	*Enterococcus faecium*	+	This study
5	8094	*Enterococcus faecium* E980	+	M de Been (3)
6	8095	*Enterococcus faecium* E1604 (EnGen0028)	+	M de Been (4)
7	8096	*Enterococcus faecium* E1861	+	M de Been (4)
8	8097	*Enterococcus faecium* E3548	+	M de Been (4)
9	8098	*Enterococcus faecium* TX1310	+	BE Murray
10	8099	*Enterococcus faecium* TX1330	+	BE Murray (2)
11	8100	*Enterococcus faecium* TX2050	+	BE Murray
12	IMT8703	*Enterococcus faecium* (K996/03-1)	+	This study
13	IMT30343	*Enterococcus faecium* (1593-5/12)	+	This study
14	IMT1318	*Enterococcus faecalis* DSM 2570 (ATCC 29212)	+	DSMZ
15	IMT4820	*Enterococcus faecalis* ATCC 14506	+	ATCC
16	IMT8076	*Enterococcus faecalis* (K37/03)	+	This study
17	IMT8081	*Enterococcus faecalis* (K417/03-5)	+	This study
18	IMT8966	*Enterococcus faecalis* (2508/03)	+	This study
19	IMT9145	*Enterococcus faecalis* (188-1/04)	+	This study
20	IMT9900	*Enterococcus faecalis* (K1055/04-4)	+	This study
21	IMT10680	*Enterococcus faecalis* (K1082/04)	+	This study
22	IMT11076	*Enterococcus faecalis* (1607/05)	+	This study
23	IMT14200	*Enterococcus faecalis* (2021/07)	+	This study
24	IMT21655	*Enterococcus faecalis* (1211-1/10)	+	This study
25	IMT10686	*Enterococcus durans* (K435/05-1)	+	This study
26	IMT38978	*Enterococcus durans*	+	This study
27	IMT4819	*Enterococcus hirae* ATCC 9790	+	ATCC
28	IMT4821	*Enterococcus hirae* ATCC 8043	+	ATCC
29	IMT38123	*Enterococcus hirae* ATCC 10541	+	ATCC

30	IMT48802	*Enterococcus hirae* (296-2/20)	+	This study
31	IMT23827	*Enterococcus raffinosus* (2019-3/10)	neg.	This study
32	IMT48810	*Enterococcus raffinosus* (276-6/20)	neg.	This study
33	IMT16264	*Enterococcus cecorum* (1492/08)	neg.	This study
34	IMT19044	*Enterococcus cecorum* (1644/09)	neg.	This study
35	IMT19051	*Enterococcus cecorum* (1645/09)	neg.	This study
36	IMT21482	*Enterococcus cecorum* (1058)	neg.	This study
37	IMT39924	*Enterococcus avium* (UW6277)	±	G Werner, RKI
38	IMT39925	*Enterococcus avium* (UW11197)	neg.	G Werner, RKI
39	IMT39926	*Enterococcus avium* (UW14640)	neg.	G Werner, RKI
40	IMT12358	*Enterococcus casseliflavus* (1860/06)	+	This study
41	IMT39927	*Enterococcus casseliflavus* (UW13404)	+	G Werner, RKI
42	IMT39928	*Enterococcus casseliflavus* (UW13463)	+	G Werner, RKI
43	IMT39929	*Enterococcus casseliflavus* (UW13577)	+	G Werner, RKI
44	IMT12257	*Enterococcus gallinarum* (L176/06-1)	+	This study
45	IMT12258	*Enterococcus gallinarum* (L176/06-2)	+	This study
46	IMT12261	*Enterococcus gallinarum* (L179/06-1)	+	This study
47	IMT12262	*Enterococcus gallinarum* (L179/06-2)	+	This study

*Abbreviations: ADI, arginine deiminase activity in Rosco Diagnostica ADH assays (+, positive reaction; neg., negative reaction; ±, variable reaction); ATCC, American Type Culture Collection; DSMZ, Deutsche Sammlung von Mikroorganismen und Zellkulturen, Braunschweig, Germany; NCIMB, National Collection of Industrial, Food and Marine Bacteria, Aberdeen Scotland, UK; RKI, Robert Koch Institute. Additional internal source or laboratory designations for isolates are indicated in parentheses.

References

1. Arduino, RC, Murray BE, Rakita RM. Roles of antibodies and complement phagocytic killing of enterococci. *Infect Immun*. 1994; 62:987-993. (TX0016, TEX16)
2. Qin, X, Galloway-Peña JR, Sillanpaa J, Roh JH, Nallapareddy SR, Chowdhury S, Bourgogne A, Choudhury T, Muzny DM, Buhay CJ, *et al*. Complete genome sequence of *Enterococcus faecium* strain TX16 and comparative genomic analysis of *Enterococcus faecium* genomes. *BMC Microbiology*. 2012; 12:135. http://www.biomedcentral.com/1471-2180/12/135.
3. van Shaik W, Top J, Riley DR, Boekhorst J, Vrijenhoek JE, Schapendonk CM, Hendrickx AP, Nijman IJ, Bonten MJ, Tettelin H, *et al*. Pyrosequencing-based comparative genome analysis of the nosocomial pathogen *Enterococcus faecium* and identification of a large transferable pathogenicity island. *BMC Genomics*. 2010; 11:239. https://doi.org/10.1186/1471-2164-11-239.
4. Lebreton, F, van Schaik W, McGuire AM, Godfrey P, Griggs A, Mazumdar V, Corander J, Cheng L, Saif S, Young S, *et al*. Emergence of epidemic multidrug-resistant *Enterococcus faecium* from animal and commensal strains. *mBio*. 2013; 4:e00534-13.
