# Supplementary material for: A virulence factor as a therapeutic: the probiotic Enterococcus faecium SF68 arginine deiminase inhibits innate immune signaling pathways
Source: Gut Microbes. 2022 Aug 3;14(1):2106105. doi: 10.1080/19490976.2022.2106105 (PMC9351580; doi:10.1080/19490976.2022.2106105)
Supplement: Supplemental Material [file KGMI_A_2106105_SM4272.zip › Manuscript Ghazisaeedi et al Suppl TableS3.pdf]

Ghazisaeedi *et al*., 2022. Supplementary Table S3

Primers used in Real-Time PCR (RT-PCR)

Gene	Primer	Sequence (5´-3´)	Reference
β-Actin	β-actinF	GGACTTCGAGCAGGAGATGG	1
	β-actinR	GCACCGTGTTGGCGTAGAGG	
IL-6	IL-6suis-for	CAGAAAACAACCTGAACCTTCC	2
	IL-6suis-rev	TCCACTCGTTCTGTGACTGC	
IL-8	poIL8F-2	TTCGATGCCAGTGCATAAATA	3
	poIL8R-2	CTGTACAACCTTCTGCACCCA	
Bax	BAX_for	GGTCGCGCTTTTCTACTTTG	4
	BAX_rev	CGATCTCGAAGGAAGTCCAG	

1. Dozois CM, Oswald E, Gautier N, Serthelon JP, Fairbrother JM, Oswald IP. A reverse transcription-polymerase chain reaction method to analyze porcine cytokine gene expression. *Vet Immunol Immunopathol*. 1997; 58:287-300.
2. Schierack P, Nordhoff M, Pollmann M, Weyrauch KD, Amasheh S, Lodemann U, Jores J, Tachu B, Kleta S, Blikslager A, et al. Characterization of a porcine intestinal epithelial cell line for *in vitro* studies of microbial pathogenesis in swine. *Histochem Cell Biol*. 2006;125:293-305.
3. Collado-Romero M, Arce C, Ramírez-Boo M, Carvajal A, Garrido JJ. Quantitative analysis of the immune response upon *Salmonella* Typhimurium infection along the porcine intestinal gut. *Vet Res*. 2010; 41:23. doi: 10.1051/vetres/2009072.
4. Yuan B, Liang S, Jin Y-X, Kwon J-W, Zhang J-B, Kim N-H. Progesterone influences cytoplasmic maturation in porcine oocytes developing *in vitro*. *Peer J*. 2016; 4:e2454.
